# *Hairiness* Gene Regulated Multicellular, Non-Glandular Trichome Formation in Pepper Species

**DOI:** 10.3389/fpls.2021.784755

**Published:** 2021-12-16

**Authors:** Jinqiu Liu, Haoran Wang, Mengmeng Liu, Jinkui Liu, Sujun Liu, Qing Cheng, Huolin Shen

**Affiliations:** Beijing Key Laboratory of Growth and Developmental Regulation for Protected Vegetable Crops, Department of Vegetable Science, College of Horticulture, China Agricultural University, Beijing, China

**Keywords:** pepper, trichomes, *Hairiness*, zinc-finger protein, promoter

## Abstract

Trichomes are unicellular or multicellular epidermal structures that play a defensive role against environmental stresses. Although unicellular trichomes have been extensively studied as a mechanistic model, the genes involved in multicellular trichome formation are not well understood. In this study, we first classified the trichome morphology structures in *Capsicum* species using 280 diverse peppers. We cloned a key gene (*Hairiness*) on chromosome 10, which mainly controlled the formation of multicellular non-glandular trichomes (types II, III, and V). *Hairiness* encodes a Cys2-His2 zinc-finger protein, and virus-induced gene silencing of the gene resulted in a hairless phenotype. Differential expression of *Hairiness* between the hairiness and hairless lines was due to variations in promoter sequences. Transgenic experiments verified the hypothesis that the promoter of *Hairiness* in the hairless line had extremely low activity causing a hairless phenotype. *Hair* controlled the formation of type I glandular trichomes in tomatoes, which was due to nucleotide differences. Taken together, our findings suggest that the regulation of multicellular trichome formation might have similar pathways, but the gene could perform slightly different functions in crops.

## Introduction

Trichomes are specialized cell structures that commonly occur in the epidermis of terrestrial plants. Trichomes can exhibit remarkable morphological variation between plant species. Trichomes can be morphologically classified based on the number of cells, or whether they are glandular or branched (Tetsuya et al., 2008; Pattanaik et al., 2014). Due to their special structure, trichomes can serve as a model to study plant cell differentiation (Johnson, 1975; Karabourniotis et al., 2010). Trichomes protect plants against biotic and abiotic stresses, arthropod herbivores, pathogens, and UV-B radiation and prevent water loss (Werker, 2000; Kennedy, 2003; Kang et al., 2014). Secondary metabolites in glandular trichomes have been utilized as food additives, in pharmaceuticals, and as natural pesticides (Aharoni et al., 2006; Schilmiller et al., 2008; Yang et al., 2015).

In *Arabidopsis*, trichomes are unicellular, dendritic, and non-glandular. Transcriptional regulation of trichome initiation in Arabidopsis involves the expression of *GLABRA1* (*GL1*), *GLABRA3* (*GL3*), *ENHANCER OF GLABRA3* (*EGL3*), and *TRANSPARENT TESTA GLABRA1* (*TTG1*). *GL3* and *EGL3* physically interact with *GL1* and *TTG1* separately, forming an MYB–bHLH–WD (MBW) complex (Jürgens, 1994; Larkin, 1994; Payne et al., 2000; Szymanski et al., 2000; Bernhardt et al., 2003). *GLABRA2* (*GL2*) encodes HD-ZIP IV transcription factors that act downstream of the complex (Ishida et al., 2007; Zhao et al., 2008). In addition, several R3 MYB regulators act as negative regulators by competing with *GL1* and interacting with *GL3/EGL3* to form an inactive complex. The inactive complex disrupts the activity of *TTG1*-*GL3*/*EGL3*-*GL1*, reduces the expression of *GL2*, and prevents neighboring cells from entering the process of trichome development (Wang et al., 2007; Wester et al., 2009; Gan et al., 2011). Furthermore, the MBW complex can be activated by Cys2-His2 (C2H2) zinc-finger proteins, such as *GIS, ZFP5, ZFP6*, and *ZFP8*, which control trichome formation on inflorescence stems and flowers (Gan et al., 2007; Zhou et al., 2011, 2013; Sun et al., 2015).

Most crops, which produce multicellular trichomes, differ from Arabidopsis in the morphological structure of trichomes. In addition, there are great differences in the morphology of trichomes among different species. For example, tobacco (*Nicotiana tabacum* L.) has many different types of trichomes, including glandular or non-glandular protection trichomes (Andersen et al., 1988). The protection trichomes have a single row to protect hair and fork hair. *Cucumis sativus* (cucumber) produces two morphologically distinct trichome types: type I glandular trichomes, which are composed of 3–15 cell bases and 4–18 celled globular glandular heads; and type II glandular trichome, which are also known as “Fruit Spines” (Chen et al., 2016). *CsGL1* and *CsGL3*, which encode HD-ZIP I and HD-ZIP VI transcription factors, are essential for the formation of type II trichomes in cucumber (Li et al., 2015; Wang et al., 2016).

Tomato (*Solanum lycopersicum* L.), a model plant of Solanaceae, has seven distinct trichome types (I–VII) (Goffreda et al., 1990), of which studies focused mostly on type I and type VI glandular trichomes (Goffreda et al., 1990; Schilmiller and Charbonneau, 2012). Type I multicellular trichome formation is controlled by *Wooly* (*Wo*), which encodes an HD-ZIP IV transcription factor, and its interactors *SlCycB2* and *Hair* (*H*), a B-type protein and a C2H2 zinc-finger protein (Yang et al., 2011; Gao et al., 2017; Chang et al., 2018). *SlMYC1* encodes bHLH transcription factors, which when knocked down do not lead to reduced density and smaller type VI glandular trichomes, and regulation of terpene biosynthesis (Xu et al., 2018). *CHALCONE ISOMERASE 1* (*CHI1*) encodes chalcone isomerase of flavonoid biosynthesis, which can reduce the density and flavonoids of type VI glandular trichomes (Kang et al., 2014).

The genus *Capsicum* produces six main trichome types (types I–VI), including three types of non-glandular (types II, III, and V) and three types of glandular trichomes (types I, IV, and VI) (Kim et al., 2012). However, there have been only a few reports on the trichome genes of pepper. The pepper trichome locus 1 (*Ptl1*) gene, which was reported in 2010, was mapped to an 80-kb BAC clone on chromosome 10. The mapping region included 14 open reading frames (ORFs); however, no candidate genes were identified (Kim et al., 2010).

In this study, we aimed to observe and categorize the trichome morphology of pepper trichomes. In addition, we used a map-based cloning approach to demonstrate that the *Hairiness* gene encodes a single C2H2 zinc-protein transcription, which controls the formation of multicellular non-glandular trichomes (types II, III, and V). Virus-induced gene silencing (VIGS) was used to determine the changes in the *Hairiness* gene that caused trichome loss in the hairiness lines. The CDS sequence of the *Hair* gene was the same between hair and hairless lines, but the promoter variation affected the *Hairiness* gene expression level and controlled the hairiness phenotype of pepper. This study could serve as a reference for further research on molecular mechanisms in pepper. We hope our findings can be useful for resistant pepper breeding and hybrid seed production.

## Materials and Methods

### Plant Materials

A total of 280 accessions used for GWAS were preserved in our laboratory (Wu et al., 2019). Two F_2_ populations, 18C2480 and 19Q6090, from crossing 18C2458 (hairless) × 18C3375 (hairiness) and 19Q6092 (hairiness) × 19C6093 (hairless), respectively, were used for rough mapping and fine mapping. F_2:3_ recombination individuals were generated from the 19Q6090 F_2_ populations.

### Scanning Electron Microscope Observation

Samples were cut into 5 × 5 mm square and fixed with 2.5% glutaraldehyde for approximately 24 h. Thereafter, the samples were washed with 0.1 M phosphate buffer (pH 7.2) and dehydrated with 50, 70, and 90% ethanol, respectively. Finally, the samples were dried in a desiccator, and a gold film of 100–150 A was plated on the surface of the sample using an E-1010 (HITACHI) ion sputtering coater. The samples were observed using the scanning electron microscope (SEM; JSM-6390/LV; https://www.jeol.co.jp).

### Trichomes Density

The young stem trichome density between the first and the second leaf and the stem between the fourth and fifth leaves, the first young leaf and the seventh leaf, calyx, and petal were dissected using a light microscope (Nikon SMZ18, https://www.nikon.com) at the 10-leaf stage. At least five individuals per line were sampled. All samples were analyzed in triplicate, and the data were expressed as the mean and SD.

### Fine Mapping of *Hairiness*

An F_2_ population (18C2450) and 12 polymorphic markers were used for rough mapping. Fine mapping was performed using the F_2_ population (19Q6090) and eight polymorphic markers. Using the F_3_ recombination individuals from the F_2_ population (19Q6090) and five InDel markers, which were determined based on the sequenced genome sequences of parent lines to determine candidate interval. The primers used are shown in Supplementary Table 6. The physical location of the markers and genes was based on the pepper genome sequence of CM334v.1.55.

### Virus-Induced Gene Silencing

Virus-induced gene silencing (VIGS) was performed according to Chung et al. (2004) with some modifications. Silence fragments were predicted using the SGN VIGS tool (https://vigs.solgenomics.net/). The constructs consisting of pTRV1, pTRV2, pTRV2-*PDS*, and pTRV2::*21340* were transformed into the vector GV3101. A mixture of cultures containing 1:1 (v:v) of pTRV1 and pTRV2 was used as a negative control, and pTRV1 and pTRV2::*PDS* were used as a reporter. Approximately 1 ml syringes without needles were used to infiltrate the cotyledons of 3-week-old seedlings. The plants inoculated with *Agrobacterium* were grown in a growth chamber with temperatures 22°C in 16 h light and 20°C in 8 h dark cycles.

### Gene Expression Analysis

The RT-PCR and real-time PCR were performed to analyze the expression levels of *Hairiness* from the hairiness line (19Q6092) and hairless line (19Q6093), respectively. RNA extraction and PCR were performed according to the method described by Liu et al. (2020). The primer sequences are shown in Supplementary Table 7.

### Promoter Sequence Variation Analysis

The 2,200 bp sequence of the ATG upstream of the hairy gene was cloned from the hairiness line (parent line 19Q6092) and hairless line (parent line 19Q6093). Homology-based cloning primers (Supplementary Table 7) were designed according to the upstream 2,200 bp sequence in the CDS database of CM334v.1.55 (https://solgenomics.net/tools/blast/). Plant CARE (http://bioinformatics.psb.ugent.be/webtools/plantcare/html/) and DNA MAN (http://www.biologydir.com/dnaman-info-1940.html) were used for sequence analysis.

### GUS Staining

The GUS assay was used to measure the promoter activity. Both promoters and short fragments were fused with the GUS reporter and inserted into the vector pCAMBIA1305.1. The primer sequences are shown in Supplementary Table 7. The constructs were transiently expressed in 4-week-old tobacco leaves, mediated by *Agrobacterium tumefaciens* strain GV3101. *A. tumefaciens* GV3101 and pCaMV35S::GUS were used as negative and positive controls, respectively. GUS staining solution (5-bromo-4-chloro-3-indolyl b-D-glucuronide, 37°C, 12 h) was used to evaluate GUS activity, and later incubated with 70% ethanol at 65°C to remove chlorophyll, before observing under a light microscope.

### Transgenic Analysis

The full-length coding sequence of *CA10g21340* from the hairiness line (19Q6092) (primers in Supplementary Table 7) was inserted into the pCAMBIA1305.1::pro*21340* (hairiness) and pCAMBIA1305.1::pro*21340* (hairless), which replaced GUS. The constructs were transformed into Micro Tom, mediated by the *A. tumefaciens* strain GV3101 (Fillatti et al., 1987).

### Statistical Analysis

All samples were analyzed in triplicates, and the data are expressed as mean ± SD unless noted otherwise. Statistical significance was determined at the 0.05 (*) and 0.01 (**) levels. All experiments were repeated at least twice with three biological replicates each time.

## Results

### Trichome Morphology and Distribution Characteristics of *Capsicum* Species

**Figure 1:**
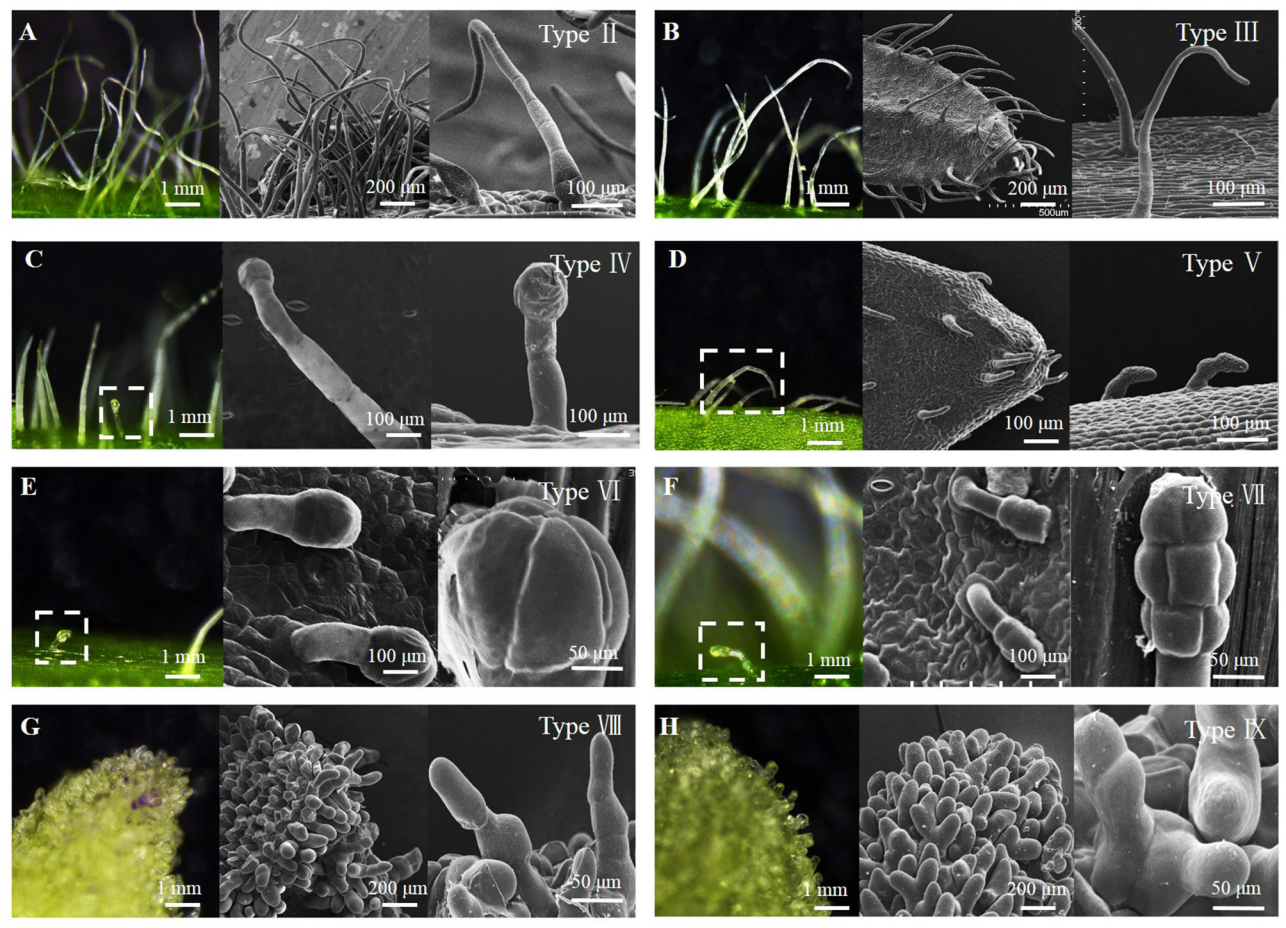
Morphological observation of pepper trichome types. **(A)** Type II, **(B)** Type III, **(C)** Type IV, **(D)** Type V, **(E)** Type VI, **(F)** Type VII, **(G)** Type VIII, and **(H)** Type IX. The observation was carried out under light microscopy and SEM.

**Figure 2:**
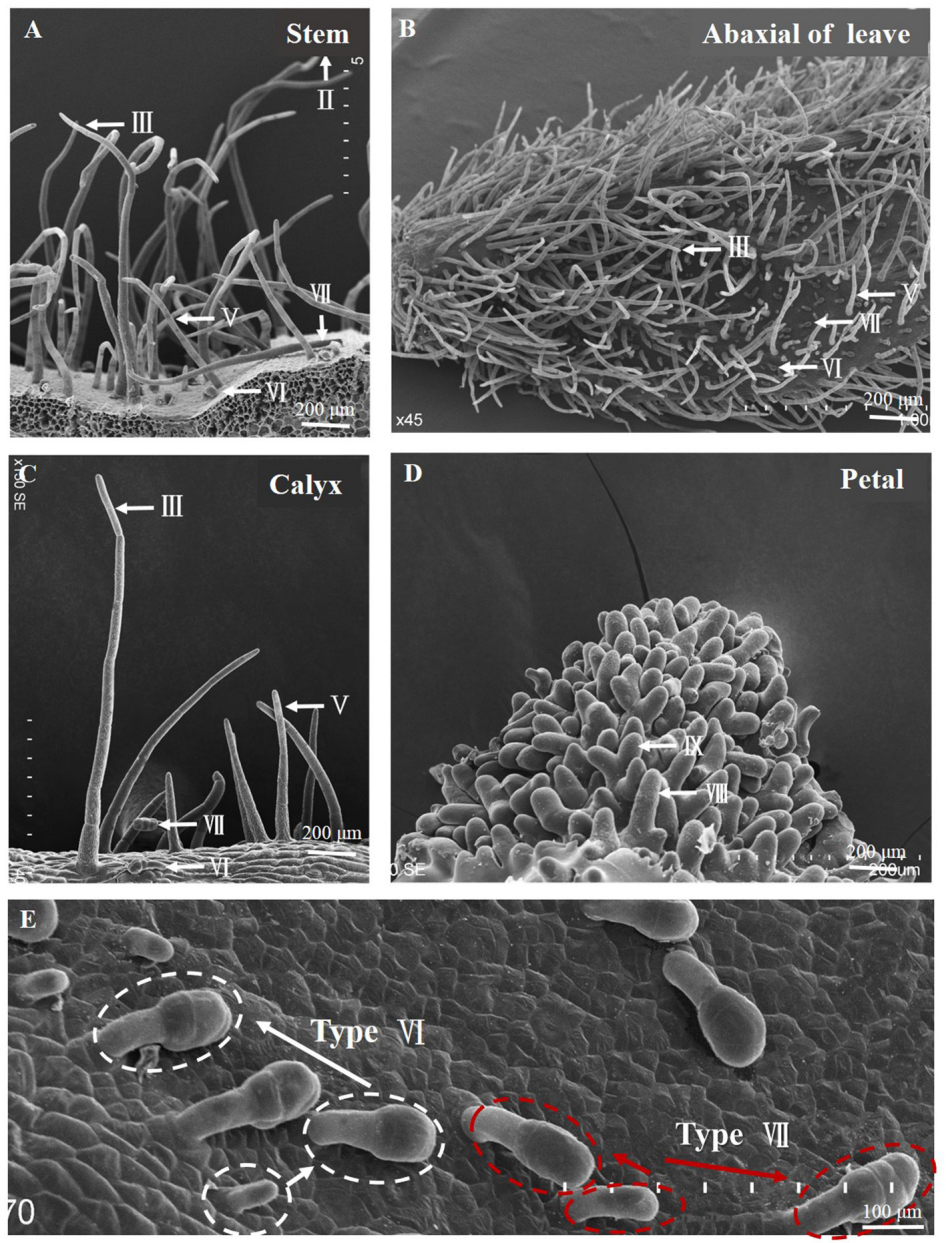
Morphological observation of pepper trichomes on different tissues. **(A)** Morphology of trichome on the stem (types II, III, V, VI, and VII). **(B)** Leaf adaxial (types III, V, VI, and VII), **(C)** Calyx (types III, V, VI, and VII), **(D)** Petal (types VIII and IX), and **(E)** Development stage of type VI and type VII.

The epidermis of stems, leaves, and flowers of 280 breeding lines of 4 pepper species (*Capsicum annuum, C*. *chinense, C*. *frutescens*, and *C*. *baccatum*) were observed for morphological identification of trichomes. There were eight types of trichomes observed, including three types of glandular trichomes (types IV, VI, and VII) and five types of non-glandular trichomes (types II, III, V, VIII, and IX) (Figure 1). Table 1 and Figures 2A–D show the trichome morphology.

**Table 1:** Morphological description of pepper trichomes.

**Type of trichomes**	**Description**	**Stalk length**	**Cell number of stalks**	**Glandular**
Type II	Thin non-glandular trichome consisting of 15–120 cells, and 2.0–12.5 mm long with a multicellular base	2.0–12.5	18–120	-
Type III	Similar to II, but with a unicellular and flat base, consisting of 3–115 cells, and 0.2–11.5 mm long	0.5–11.5	3–116	-
Type IV	Thin glandular trichome consisting of 2–14 cells, 0.1–10.3 mm long, unicellular base, and a small and round glandular cell in the trichome tip	0.1–10.3	2–14	1
Type V	Similar to type III but shorter with 3 cells, and 0.1–10.17 mm long. External walls clear intercellular sections	0.1–10.2	3–14	-
Type VI	Thick and short glandular trichome consisting of 1 stalk cell and a head made up of 6 cells. About 0.05–10.065 mm long and the shape of glandular cells are near-spherical	0.05–10.065	1	6
Type VII	Similar to type VI, 0.065–10.08 mm long but glandular head is made up of 8 cells and near-elliptical	0.065–10.08	1	8
Type VIII	Shorter than type V, with 3–14 cells and 0.07–10.10 mm long. The diameter of stalk cells is approximately two times that of type V, and the cells are irregularly arranged	0.07–1.10	3–14	-
Type IX	Similar to type VIII in cell shape and length. Forked hair, with each branch consisting of 1–13 cells	0.05–10.10	4–16	-

Based on the statistics of the number of trichomes in different tissues of 280 lines, referring to Luckwill's classification of trichome density of *Lycopersicon* (Luckwill, 1943), pepper trichomes were divided into four grades: abundant (800–1,500 number/cm^2^), medium (200–800 number/cm^2^), sparse (1–200 number/cm^2^), and no (0 trichomes). Among the observed pepper species, 118 lines were hairless; 106 lines had trichomes on young stems and young leaves (8 sparse, 95 medium, and 3 abundant trichome lines); 40 lines had trichomes that occurred only on leaves (3 sparse, 30 medium, and 7 abundant trichome lines); and 16 lines showed abundant trichomes on all organs (Supplementary Table 1).

### Phenotypic Analysis of Trichomes

**Figure 3:**
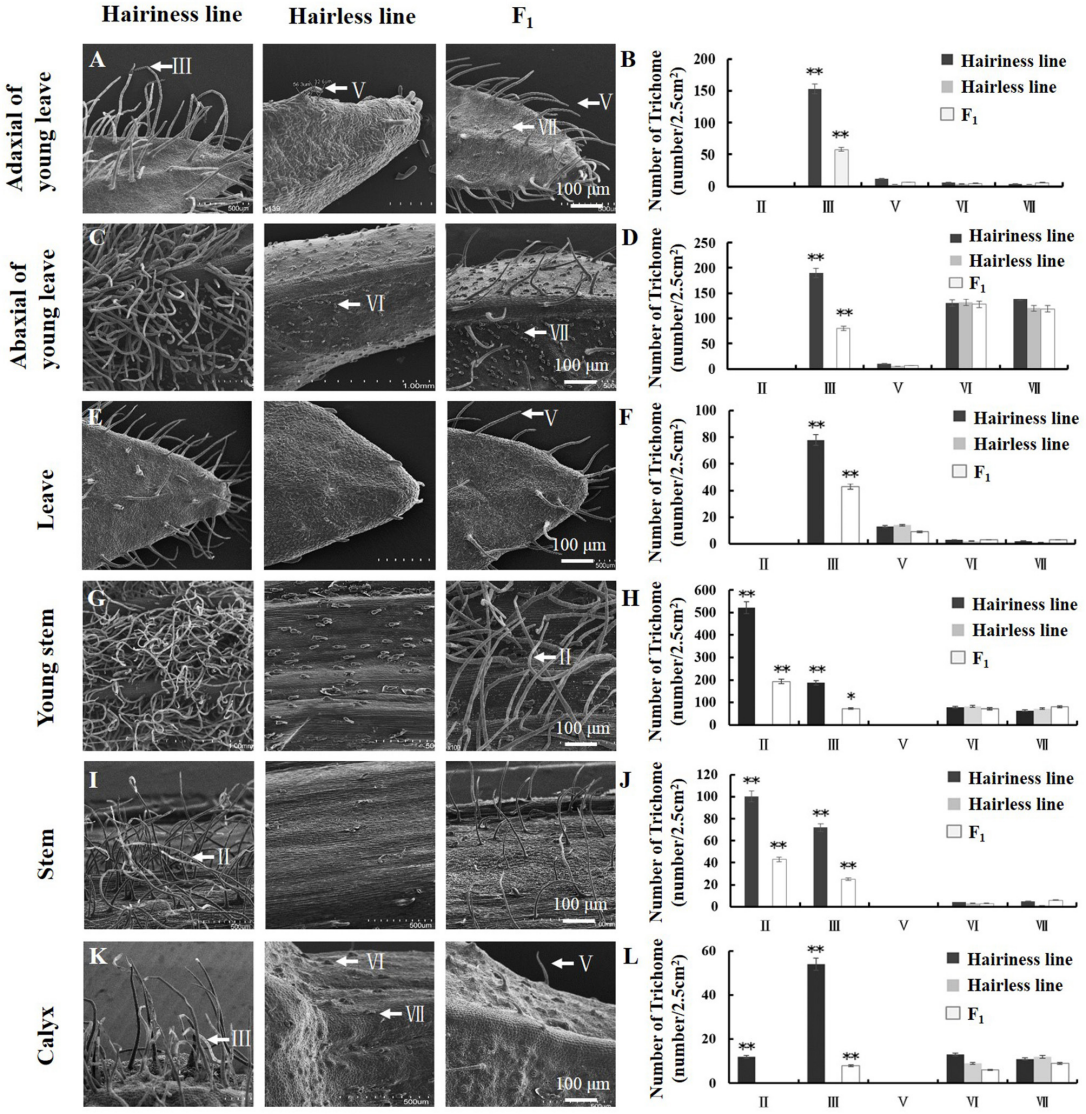
Trichome phenotypes and statistics of parent and F_1_ generation lines used for fine mapping. **(A,B)** Type and the number of trichomes on adaxial of young leaves. **(C,D)** Type and the number of trichomes on abaxial of young leaves. **(E,F)** Type and number of trichomes on leaves. **(G,H)** Type and the number of trichomes on the young stem. **(I,J)** Type and the number of trichomes on the stem. **(K,L)** Type and the number of trichomes on the calyx. SEM observation of trichomes and the arrows show types of trichomes. Error bars represent SD (*n* = 3). **p < 0.01, *p < 0.05.

We examined trichome phenotypes on the stems, leaves, and calyxes of the hairless inbred line “19Q6093,” hairiness inbred line “19Q6092” (CM334), and F_1_ progeny (Figures 3A,C,E,G,I,K). Hairiness line had four types of trichomes (types II, III, VI, and VII) on young stems, stems, and calyxes; the numbers of these trichomes were 521, 187, 78, and 63 gen/cm^2^; 100, 72, 4, and 5 gen/cm^2^; and 12, 54, 13, and 11 gen/cm^2^, respectively. The numbers of these trichomes (types III, V, VI, and VII) on the adaxial side of young leaves, abaxial side of young leaves, and leaves were 153, 12, 6, and 4 gen/cm^2^; 190, 10, 130, and 142 gen/cm^2^; and 78, 12, 3, and 2 gen/cm^2^, respectively. Hairless lines and F_1_ progeny were counted using the same statistical method as for the number of trichomes (Figures 3B,D,F,H,J,L).

By phenotypic comparison, type VI and VII trichomes were distributed on the hairiness line, hairless line, and F_1_ plants, with no significant difference in the number of trichomes among them. However, the number of type III trichomes on young leaves and leaves of hairiness line were 2.5- to 13-fold and 2-fold higher, respectively; the number of type II and III trichomes on hair line stems was 2- to 13-fold higher; and the number of type III trichomes on hair line calyxes was 7-fold higher than that on F_1_ progeny, whereas the hairless lines had no trichomes. These results suggest that the *Hairiness* gene in this study mainly controlled the formation of multicellular non-glandular trichomes (type II, III, and V) in pepper.

The phenotype of the F_2_ populations (18C2480 and 19Q6090) was consistent with that of the hairiness, F_1_ progeny, and hairless lines, with the number of plants 175, 335, and 165, respectively, for 18C2480 and 220, 429, and 220, respectively, for 19Q6090 F_2_ populations. Both were in good agreement with the expected 3:1 Mendelian ratio (χ^2^ = 0.862, *p* > 0.05; χ^2^ = 0.923, *p* > 0.05) (Onchiri, 2013) (Supplementary Table 2). These results suggest the presence of a single dominant locus responsible for the non-glandular trichome trait in the tested populations.

### Fine Mapping of *Hairiness*

*Hairiness* parent lines (19Q6092 and 6903) were screened using 350 SSR markers, which covered the whole genome of pepper. A total of 21 polymorphic markers were obtained; with both markers SR324 and SR519 located on the same side of chromosome 10 of the *Hairiness* gene.

**Figure 4:**
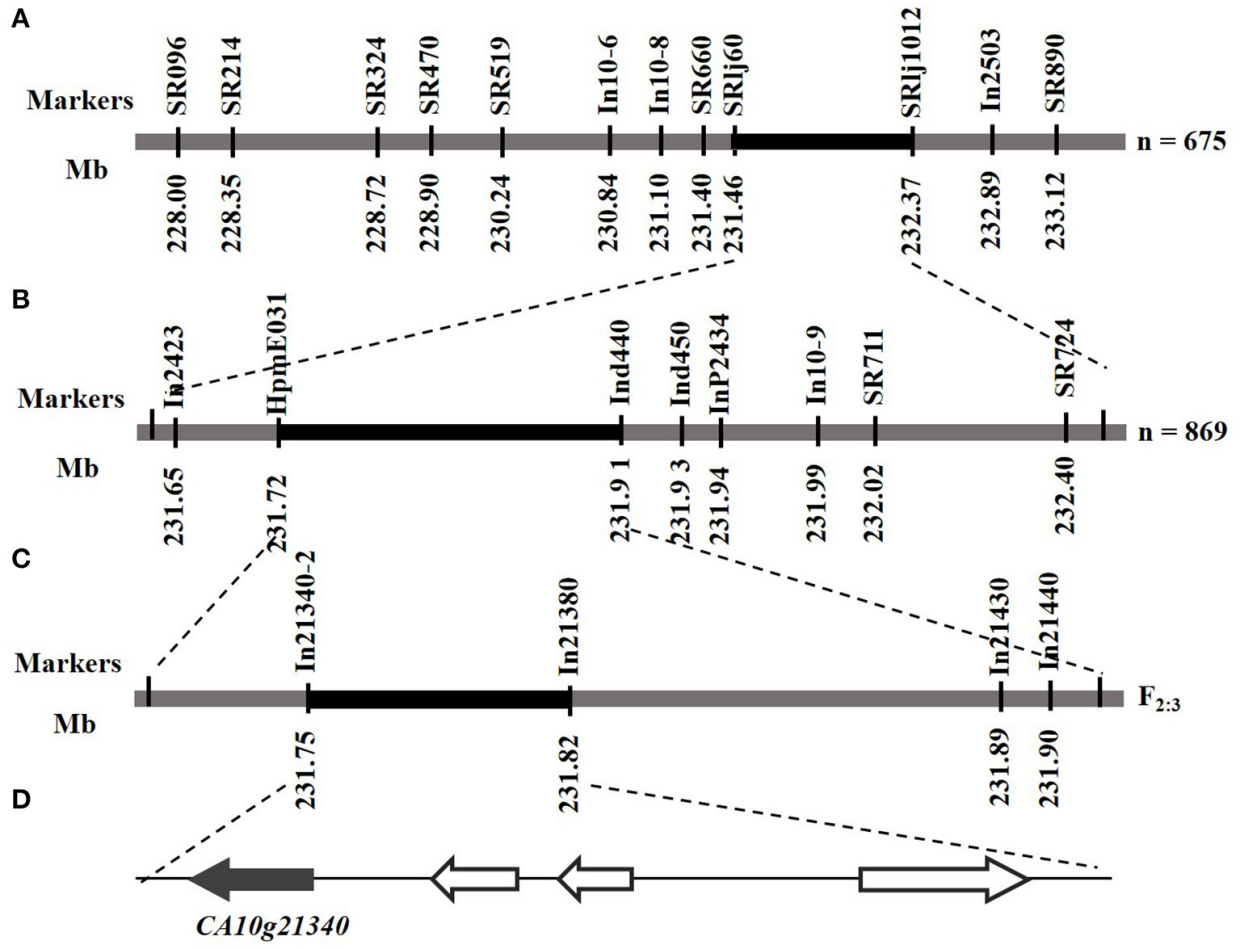
Genetic and physical maps of the *Hairiness* gene and candidate gene analysis. **(A)** Rough mapping of *Hairiness*, where *n* represents F_2_ population from the cross of 18C2458 (hairless) × 18C3375 (hairiness). Based on 12 markers and 675 F_2_ individuals, *Hairiness* was delimited between the marker SRlj60 and SRlj1012. **(B)** Fine mapping of *Hairiness*; *Hairiness* was mapped in a 176-kb region between markers HpmE031 and Ind440 with 869 F_2_ individuals (19Q6090) from the cross of 19Q6902 (hairiness) × 19Q6093 (hairless). **(C)** F_3_ exchange individuals from F_2_ individuals (19Q6090) to reduce the location region; positions of the markers are indicated in 70 kb region between markers In21340-2 and In21380. **(D)** The location of annotation genes in the target region, according to the CM334 genomics network (https://solgenomics.net/).

Twelve polymorphic markers were used to screen the F_2_ population (18C2480, *n* = 657) to lock the genetic interval for *Hairiness* (Figure 4A), and the locus was delimited by SRlj60 and SRlj1012. A total of 869 F_2_ individuals (19Q6090) and eight markers (HpmE031, SR711, SR724, In2423, Ind440, Ind450, InP2434, and In10-9) were used to narrow the target locus. The *Hairiness* gene was delimited to the region between HpmE031 and Ind440 (Figure 4B). According to the pepper reference genome sequence, the physical distance was approximately 176.62 kb. In addition, we identified five individuals with recombination near HpmE031 and Ind440. Using the F_3_ recombination individuals (Supplementary Table 3) and five InDel markers (Ind21340-1, In21380, Ind21430, and In21440-1) delimited the Hairiness trait for a 70-kb region, which encompassed four annotation genes (Figures 4C,D and Supplementary Table 4).

### Identifying Candidate Genes

To determine which was *Hairiness*, we sequenced and analyzed the expression levels of the four annotation genes in the two parental lines. DNA sequences of four annotation genes were consistent, and only *CA10g21340* showed higher expression levels in hairiness lines than in hairless lines (Supplementary Figure 1). Thus, *CA10g21340* is a likely candidate gene for *Hairiness*.

**Figure 5:**
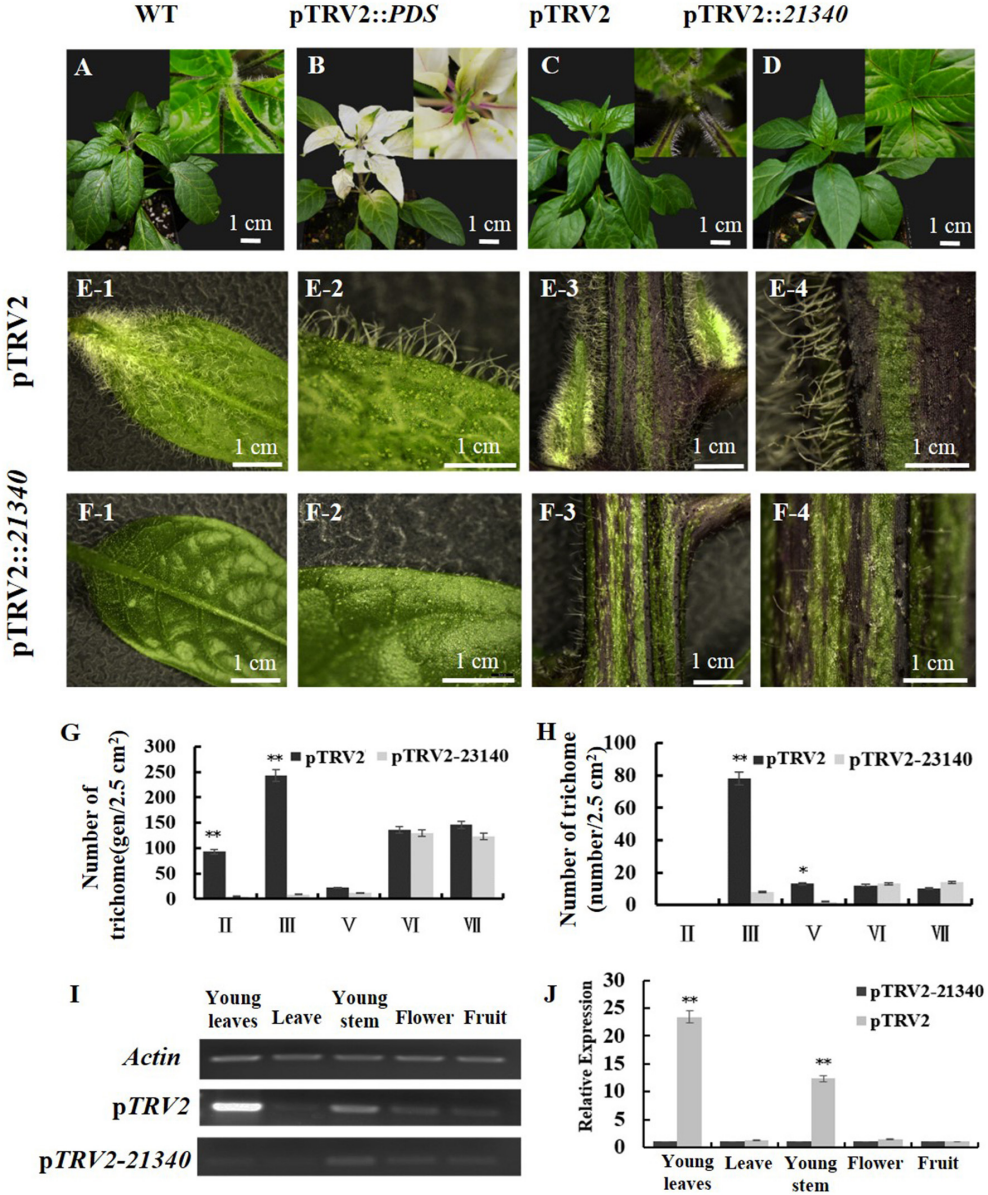
The phenotype of VIGS-treated plants. **(A)** Genotype hairiness line (CM334). **(B)** Expression of *PDS* in CM334, the plant showed photo-bleached phenotype. **(C)** and **(E1–E4)** Expression of pTRV2 in CM334, and phenotype of the plants are similar to WT. **(D)** and **(F1–F4)** Expression of pTRV2-*21340* in CM334, and decreased trichomes of those plants. **(G)** Statistics of trichomes on leaves between pTRV2 and pTRV2-*21340* infiltrated plants. **(H)** Statistics of trichomes on stems between WT and pTRV2-*21340* infiltrated plants. **(I,J)** The relative expression level of *CA10g21340* in differently infiltrated plants. Error bars represent the SD (*n* = 3). **p < 0.01, *p < 0.05. Reference gene: *UIB-3* (*Capana06g002873*).

To confirm the function of *CA10g21340*, VIGS was applied to pepper plants. Compared with the negative control (Figures 5A,C,E,G), the silenced plants showed a hairless phenotype (Figures 5B,D,F,H), and the number of types II and III non-glandular trichomes on the leaves and stems of TRV1 + TRV2::*CA10g21340* infiltrated plants decreased significantly, and the number of type V non-glandular trichomes decreased (Figures 5G,H). The expression levels of *CA10g21340* in the young leaves and young stems of TRV1 + TRV2::*CA10g21340* infiltrated plants were almost 5 and 8% of that of the negative control (Figures 5I,J). Therefore, it could be presumed that *CA10g21340* was the *Hairiness* gene that underlies the hairy phenotype.

### *Hairiness* Encodes a C2H2 Zinc-Finger Protein

*CA10g21340* is predicted to have two exons and one intron, which encodes a putative C2H2 domain. We evaluated the phylogenetic relationship of *Hairiness* with several members from *Arabidopsis* and tomato. Phylogenetic analysis revealed that *Hairiness* is closely related to the *H* (*Solyc10g078970*) and *GIS* (AT3G58070.1) genes in tomato and *Arabidopsis* (Supplementary Figure 2). *The H* gene promoted the formation of type I multicellular trichomes, and *GIS* controlled the trichome formation of inflorescence stems and flowers.

### Promoter Sequence Variation of Hairiness and Hairless Lines

**Figure 6:**
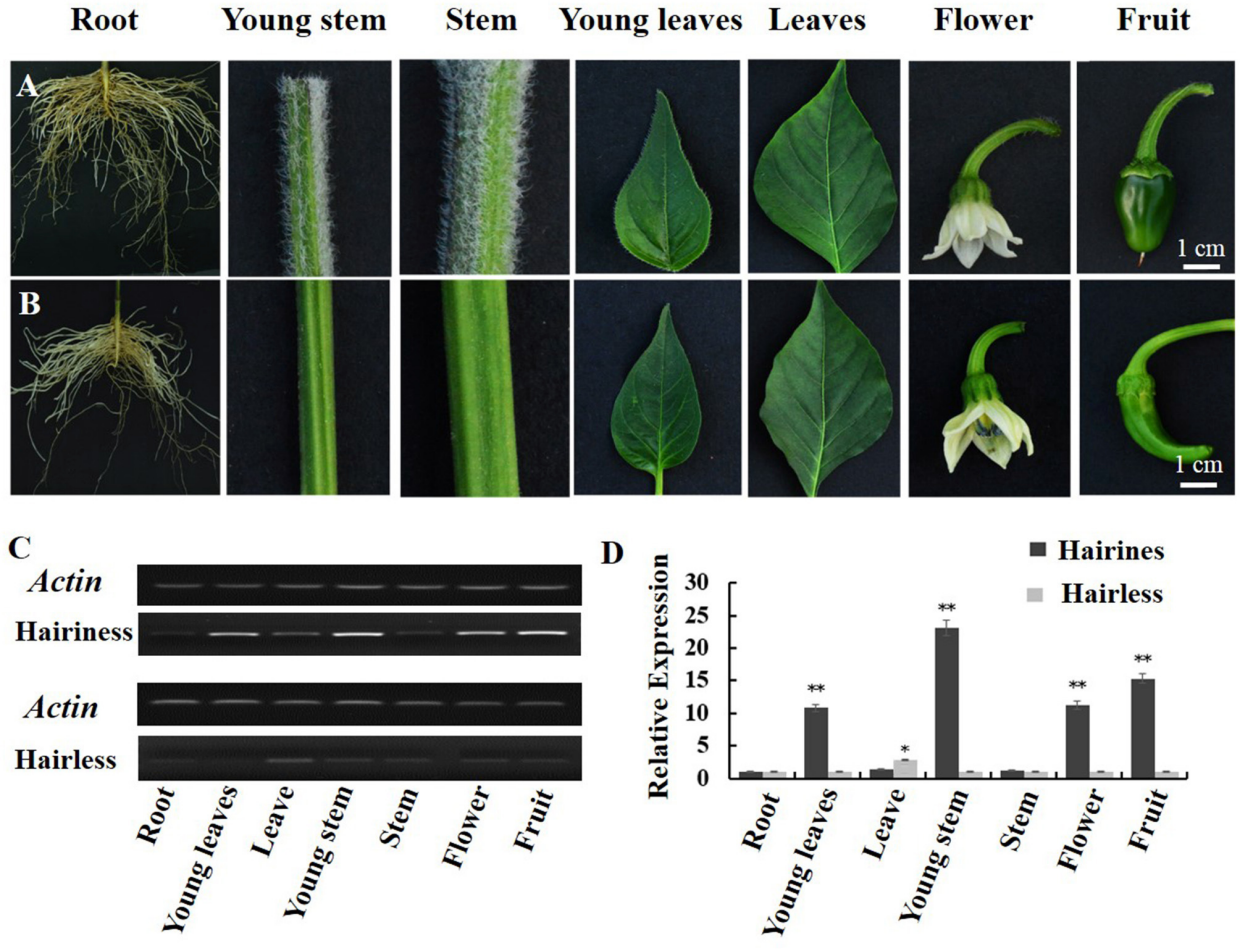
Relative expression levels of *CA10g21340* in different tissues of hairiness and hairless lines. **(A)** Organ genotypes of hairiness lines (root, young stem, stem, young leaves, leaves, flower, and fruit). **(B)** Organ genotypes of hairless lines. **(C,D)** RT-PCR and real-time PCR analysis of *CA10g21340*. Error bars represent the SD (*n* = 3). **p < 0.01, *p < 0.05. Reference gene: *UIB-3* (*Capana06g002873*).

Differential expression of *Hairiness* was observed in hairiness and hairless lines. *Hairiness* expression levels were detected in seven organs of the two parental lines (Figures 6A,B). Compared with hairless lines, the expression of *Hairiness* in young stems, young leaves, leaves, flowers, and fruits of hairiness lines increased significantly (Figures 6C,D).

**Figure 7:**
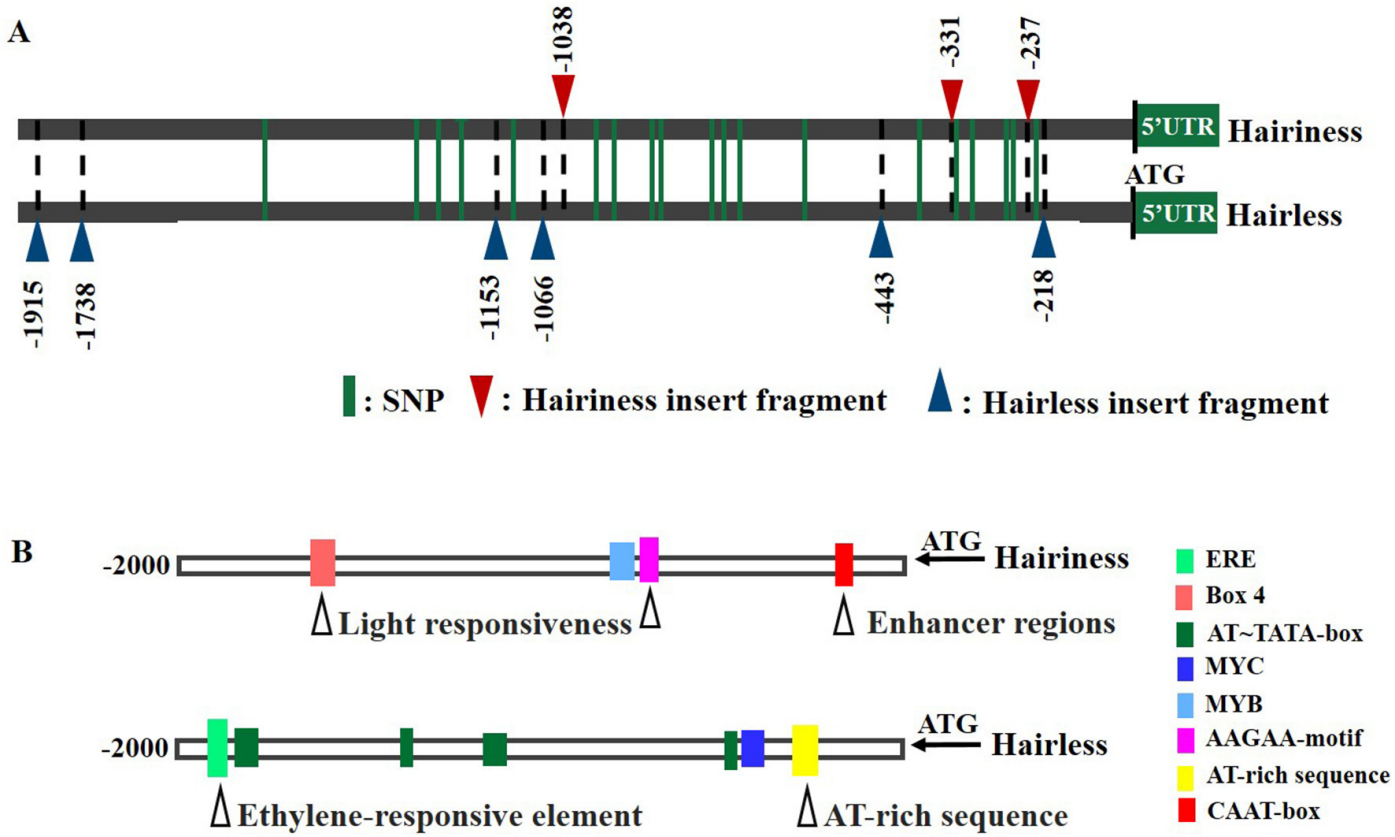
Promoter sequence differences of *CA10g21340* between hairiness and hairless lines. **(A)** A representation of promoter sequences of hairiness and hairless lines. Green lines represent a single nucleotide polymorphism. The red triangle represents the hairiness line insert fragment; the Blue triangle represents the hairless line insert fragment. **(B)** Promoter region *cis*-acting element differences between hairiness and hairless lines. The black triangle represents a variation of the *cis*-acting element.

To investigate the reason for differential expression of *Hairiness* in hairiness and hairless lines, an approximately 2.3 kb sequence upstream of ATG was amplified from these two lines. There were 4 large InDels, 2 small InDels, 19 SNPs, and no sequence variation in the 5 UTR (121 bp) (Figure 7A and Supplementary Figure 3).

However, the *cis*-acting elements of hairiness and hairless line promoter sequences, when analyzed using the PlantCARE database (Figure 7B). The hairiness promoter had one more Box-4 (light response element), MYB (stress response element), CAAT box, and AAGAA-motif (other response elements), but lacked one ERE (hormone response element), one MYC (stress response element), one AT-rich sequence, and five AT-TATA-boxes (other response elements) (Supplementary Table 5) as compared to the hairless promoter.

### Differential Expression of *Hairiness* Is Caused by Promoter Sequence Variation

**Figure 8:**
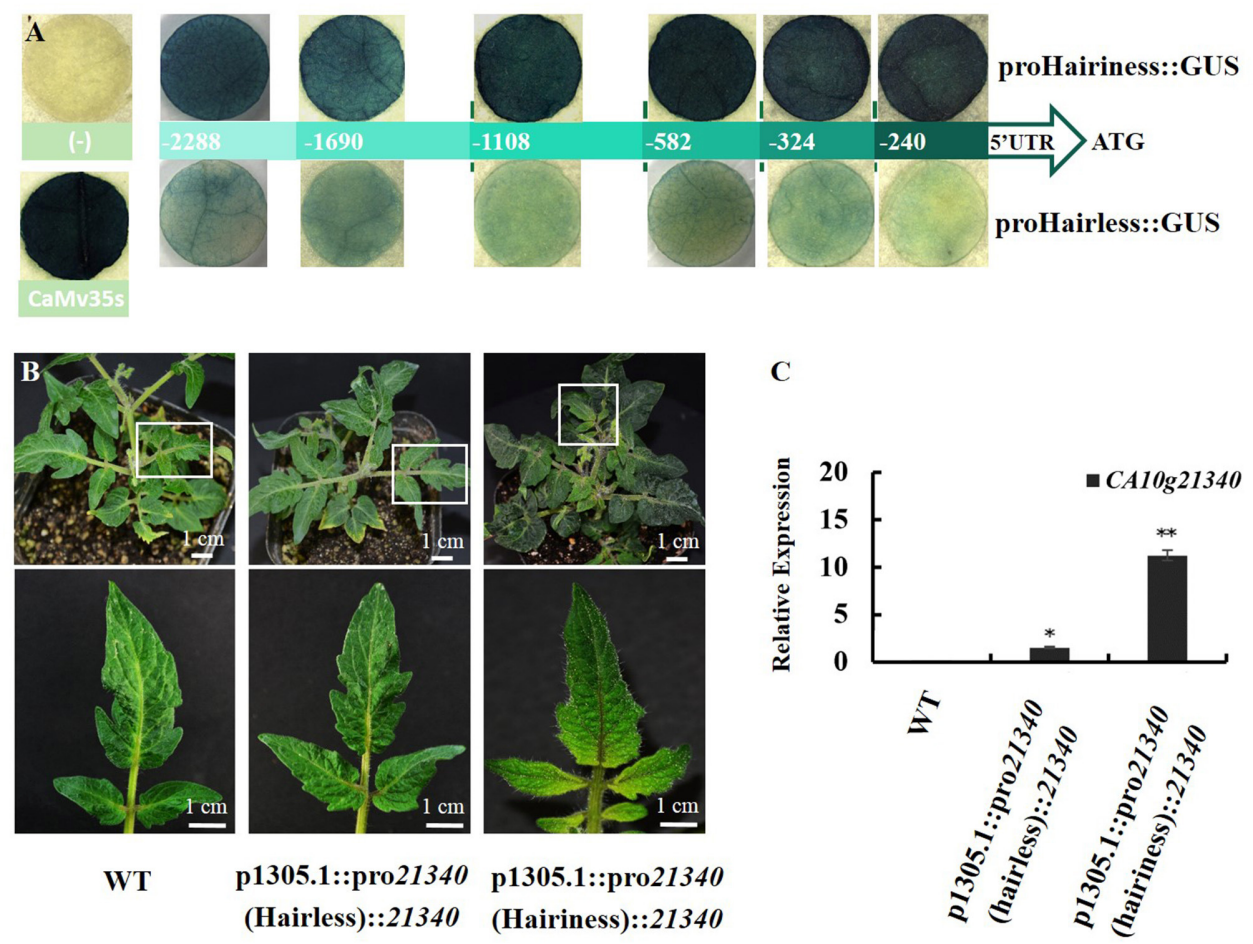
Comparative analysis of *CA10g21340* promoter activities and genetic transformation of tomato phenotype. **(A)** GUS activity in leaves of *Nicotiana benthamiana* transiently expressing promoter short fragments between hairiness and hairless line insertion pCAMBIA1305.1. **(B)** Genetic transformation of tomato phenotype: *CA10g21340* gene driven by pro*21340* (Hairiness) and pro*21340* (Hairless) in Micro Tom. **(C)** Expression analysis of *CA10g21340* with transformation tomato. Reference gene: *Actin (Solyc11g005330)*.

Both promoters and their five short fragments, from ATG to upstream −1690, −1108, −582, −324, and −240 bp were separately fused with GUS to determine their activities. The GUS activity in tobacco showed that both promoters had promoter activity, but the promoter activity of *Hairiness* from hairless lines was significantly lower than that of hairiness lines. The GUS activity of the short fragments of promoters in the hairless line showed weak light blue, whereas, in the hairiness line, it was dark-blue to blue-black. The activity of the *Hairiness* gene promoter in the hairiness line was significantly higher than that in the hairless line (Figure 8A). These results suggest that promoter sequence diversity might lead to activity differences, resulting in the differential expression patterns in hairiness and hairless lines.

Promoter activities were compared by connecting the two promoters with CDS of *Hairiness*. Two constructs, proHairiness::CDS and proHairless::CDS, were developed to perform transient transformation experiments. There was no significant difference in the number of trichomes between *Hairiness* driven by the promoter from hairless line and control, whereas the number of trichomes was considerably higher when *Hairiness* was driven by the hairiness line than by the hairless line. The number of types III, V, and VI trichomes on the leaves of hairiness line promoter transgenic tomato was 3- to 4-fold higher than that in the other two (Figure 8B and Supplementary Figure 4). In addition, the expression levels of *CA10g21340* in transgenic plants with hairiness promoter and hairless promoter were approximately 12 and 2 times higher, respectively, than those in control plants (Figure 8C). These results suggest that differential expression of *Hairiness* between hairiness and hairless lines might be caused by the variations in the gene promoter sequence, which in turn caused the hairless phenotype.

## Discussion

### *Hairiness* Triggered Non-Glandular Trichome Formation in Pepper

Morphology of unicellular trichomes of *Arabidopsis thaliana* (Tetsuya et al., 2008; Pattanaik et al., 2014), and multicellular trichomes of tomato (Luckwill, 1943) and cucumber (Chen et al., 2014) are well-studied. In addition, a previous study had reported on the morphology of pepper trichomes (Kim et al., 2012).

Through the observation, statistics, and morphological description of the trichomes of 280 pepper species (Figure 1, Table 1), trichomes can be divided into eight types (types II–IX) (Figure 1). According to the presence/absence of glandular cells, they could be classified into three (types IV, VI, and VII) glandular trichome and five (types II, III, V, VIII, and IX) non-glandular trichome types. Only one previous study (Kim et al., 2012) has reported on the classification of pepper trichomes, including five pepper species (*C. annuum, C*. *chinense, C*. *frutescens, C*. *pubescens*, and *C*. *baccatum*). In the present study, we investigated the trichomes of stems and leaves and reported six main trichome types (types I–V and VII), including three types of non-glandular trichomes (types II, III, and V) and three types of glandular trichomes (types I, IV, and VII).

Compared with the results of our study, the differences of glandular trichomes were types I and VII. Type I trichomes contained a long (~2 mm) multicellular stalk, a gland cell at the top, which occurred only on the stem and leaves of *C*. *chinensis*. Our investigation included four lines of *C*. *chinensis*, all of which were hairless phenotypes, and no type I was observed. Type VI and VII glandular trichomes were regarded as the same type because both had a similar structure and length (less than 0.1 mm) (Figure 1, Table 1). However, at the late stage of trichome development, the differentiation of glandular cells was significantly different in the morphology and the numbers of Type VI and VII glandular trichomes, as observed under the electron microscope (Figure 2E). Therefore, they can be classified as type VI and VII glandular cells. The differences in non-glandular trichomes, types VIII and IX, were only observed at the top edge of petals, were shorter (<0.1 mm), and the stalk cell was larger and rounder than other trichome cells. Since Kim did not observe the trichomes of flower organs, non-glandular trichomes of types VIII and IX were not reported. Overall, there were nine trichome types (types II–IX) of pepper, and based on the presence/absence of glandular cells, they can be classified into four glandular trichomes (types I, IV, VI, and VII) and five non-glandular trichomes (types II, III, V, VIII, and IX).

Phenotypic statistics are key to gene mapping. Based on the investigation of hair-type parental lines, F_1_ generation, and F_2_ population, we determined that the hairiness phenotype referred to non-glandular trichomes of types II, III, and V.

### *CA10g21340* Is a Strong Candidate Gene of *Hairiness*

Several genes have been identified as potential candidates for known multicellular trichome formation in tomato, cucumber, and tobacco through genetic mapping (Rick and Butler, 1956; Serna and Martin, 2006; Yang et al., 2014; Lashbrooke et al., 2015; Li et al., 2015; Zhao et al., 2015; Cui et al., 2016). However, genes related to trichome formation have been poorly studied in pepper plants. In 2004, Du Feng used a hairless line (02090) and hairiness line (02091) and by segregation population, genetic analysis showed that the hairiness trait of pepper was controlled by a single dominant locus. Random amplification of polymorphic DNA (RAPD) analysis of the F_2_ population was carried out using the BSA method. The hairiness exchange value of RAPD marker S1513_630_ was 3.97%, and the genetic distance was 3.98 cM; however, no follow-up tests were carried out (Du, 2004). In 2010, Kim constructed an F_2_ population by screening a pepper BAC library and using a hairiness line (CM334) and hairless line (*Chilsungcho* cultivar). The *Ptl1* gene was mapped to the physical distance of 23.9 kb between markers HpmE031 and Tco on chromosome 10, in which there were 14 ORFs, but no candidate gene was identified (Kim et al., 2010). We blasted the markers HpmE031 and Tco, which are closely linked to *Ptl1* in CM334v.1.55. The mapping interval was located on 231711985–231736354 of chromosome 10. In the present study, the *Hairiness* gene was also located by mapping-based cloning, using the marker HpmE031 to locate *Hairiness* in 231711985–231900189 of chromosome 10 between HpmE031 and Ind440, which included the location interval reported by Kim. We further reduced the mapping interval to 231754268–231815719 by using F_3_ exchange individuals and four InDel markers, which was approximately 20 kb downstream of that reported by Kim.

Four annotated genes were located in the 70-kb region of *Hairiness*, and we found that *Ca10g21340* had the highest homology with *GIS* of *Arabidopsis* and *H* of tomato, which encoded C2H2 zinc-finger protein. *GIS* (*AT3G58070*) regulates the formation (An et al., 2012a) and branch cells of trichomes (An et al., 2012b) on the inflorescence of main stems, which is involved in regulating the transformation of developmental stages. *H* (*Solyc10g078970*) was involved in the multicellular trichome formation of types I and VI, which regulated the development of epidermal cells. The C2H2 zinc-finger domain is conserved in monocotyledons and dicotyledons and may have similar functions. In *Arabidopsis, ZFP5* regulates the formation of trichomes on flowers, leaves, branches, and main stem inflorescences, whereas *ZFP6* promotes the growth of trichomes on flowers and main stems (Gan et al., 2007; Zhou et al., 2011, 2013; Sun et al., 2015). Therefore, *CA10g21340* might be a candidate gene for controlling hairiness formation in pepper plants. The expression of the VIGS suppressor gene in hairiness lines could significantly reduce the number of hairy plants, which further confirmed *CA10g21340* as a strong candidate for the pepper hairiness gene.

In addition, in this study, transient expression in tobacco proved that the variation in promoter sequences led to a low expression of *Hairiness* in hairless lines, which could not form multicellular non-glandular trichomes. In tomato, *H* controlled the formation of type I glandular trichomes of tomato, which had 45% homology with *Hairiness* in pepper (Chang et al., 2018). Overexpression of *Hairiness* in tomatoes caused significant increases in trichome density. The findings of this study suggest that the regulation of multicellular trichome formation might have similar pathways, but the gene could perform slightly different functions in crops.

## Data Availability Statement

The original contributions presented in the study are included in the article/Supplementary Material, further inquiries can be directed to the corresponding author/s.

## Author Contributions

JinqL, ML, and HW planned and designed the experiments. JinqL, JinkL, and SL phenotypic were observed and imaged by SEM. HW, JinqL, and ML planted and maintained field experiments. JinqL, ML, and QC evaluated the genetic transformation of tomatoes. HW, JinkL, and JinqL wrote the manuscript. All authors contributed to the article and approved the submitted version.

## Funding

This study was supported by the National Key Research and Development Program of China (2017YFD0101903), the Beijing Fruit Vegetables Innovation Team of Modern Agricultural Industry Technology System (BAIC01-2021), and the Construction of Beijing Science and Technology Innovation and Service Capacity in Top Subjects (CEFF-PXM2019_014207_000032).

## Conflict of Interest

The authors declare that the research was conducted in the absence of any commercial or financial relationships that could be construed as a potential conflict of interest.

## Publisher's Note

All claims expressed in this article are solely those of the authors and do not necessarily represent those of their affiliated organizations, or those of the publisher, the editors and the reviewers. Any product that may be evaluated in this article, or claim that may be made by its manufacturer, is not guaranteed or endorsed by the publisher.
