# Supplementary material for: Hairiness Gene Regulated Multicellular, Non-Glandular Trichome Formation in Pepper Species
Source: Front Plant Sci. 2021 Dec 16;12:784755. doi: 10.3389/fpls.2021.784755 (PMC8716684; doi:10.3389/fpls.2021.784755)
Supplement: Supplementary file 1 [file Data_Sheet_1.docx]

Supplementary information for

***Hairiness* gene regulated multicellular, non-glandular trichomes formation in pepper**

Jinqiu Liu^1^, Haoran Wang^1^, Mengmeng Liu^1^, Jinkui Liu^1^, Yihao Wang^1^, Qing Cheng^1^, Huolin Shen^1*^

***Supplementary Figure***

**Supplementary Figure 1** Sequence alignment and expression analysis of annotation genes (**A**) Nucleotide sequence alignment of *CA10g21340*. **(B)** Expression level of annotation genes

**Supplementary Figure 2** Evolutionary analysis of annotation genes (**A)** Domain analysis of *CA10g21340*. **(B)** Phylogenetic tree analysis of C2H2 proteins

**Supplementary Figure 3** Sequence alignments of *CA10g21340* on 5’UTR region between hairiness line and hairless line

**Supplementary Figure 4** Trichome phenotypes and statistics of transgenic tomato. (**A**) Trichome phenotype of WT. **(B)** Trichome phenotype of *CA10g21340* gene driven by pro*21340* (Hairless) in Micro Tom. **(C)** Trichome phenotype of *CA10g21340* gene driven by pro*21340* (Hairiness) in Micro Tom. **(D)** Type and number of trichomes on leaves of WT and transgenic tomato.***Supplementary Table***

**Supplementary Table 1 Trichomes type and density of 280 pepper**

**Supplementary Table 2 Genetic analysis of Hairiness gene**

**Supplementary Table 3 Genotypes and phenotypes of F3 exchange individuals**

**Supplementary Table 4 Annotated genes in the mapping region**

**Supplementary Table 5 Cis-acting elements in the CA10g21340 promoter regions**

**Supplementary Table 6 Information of InDel markers in fine mapping of Hairiness gene**

**Supplementary Table 7 Information of primers used to develop constructs and qPCR of Hairiness gene**

**
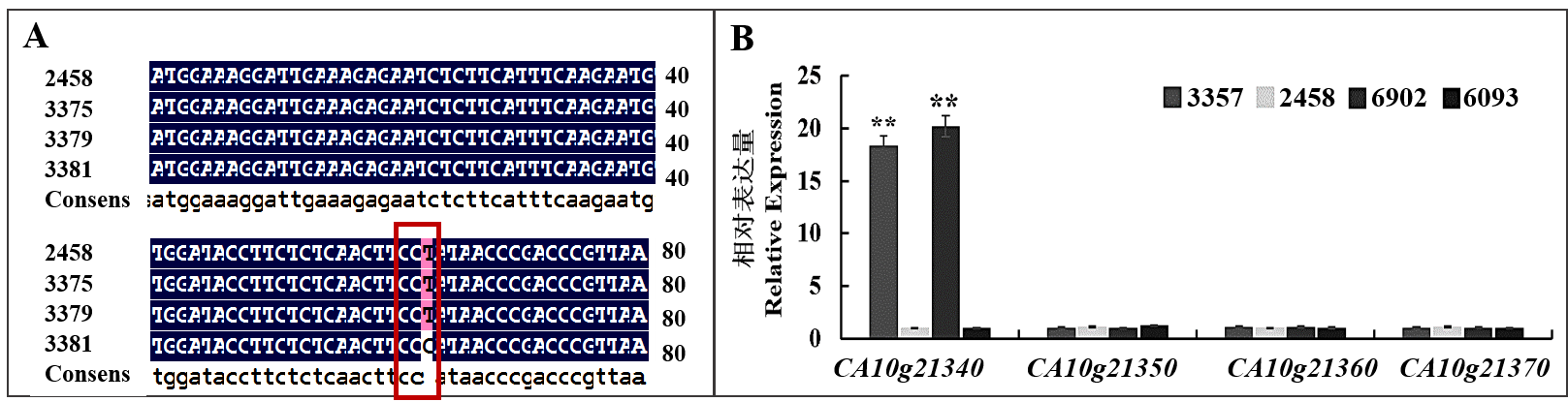
Supplementary Figures**

**Supplementary Figure 1** Sequence alignment and expression analysis of annotation genes (**A)** Nucleotide sequence alignment of *CA10g21340*. (**B)** Expression level of annotation genes

**
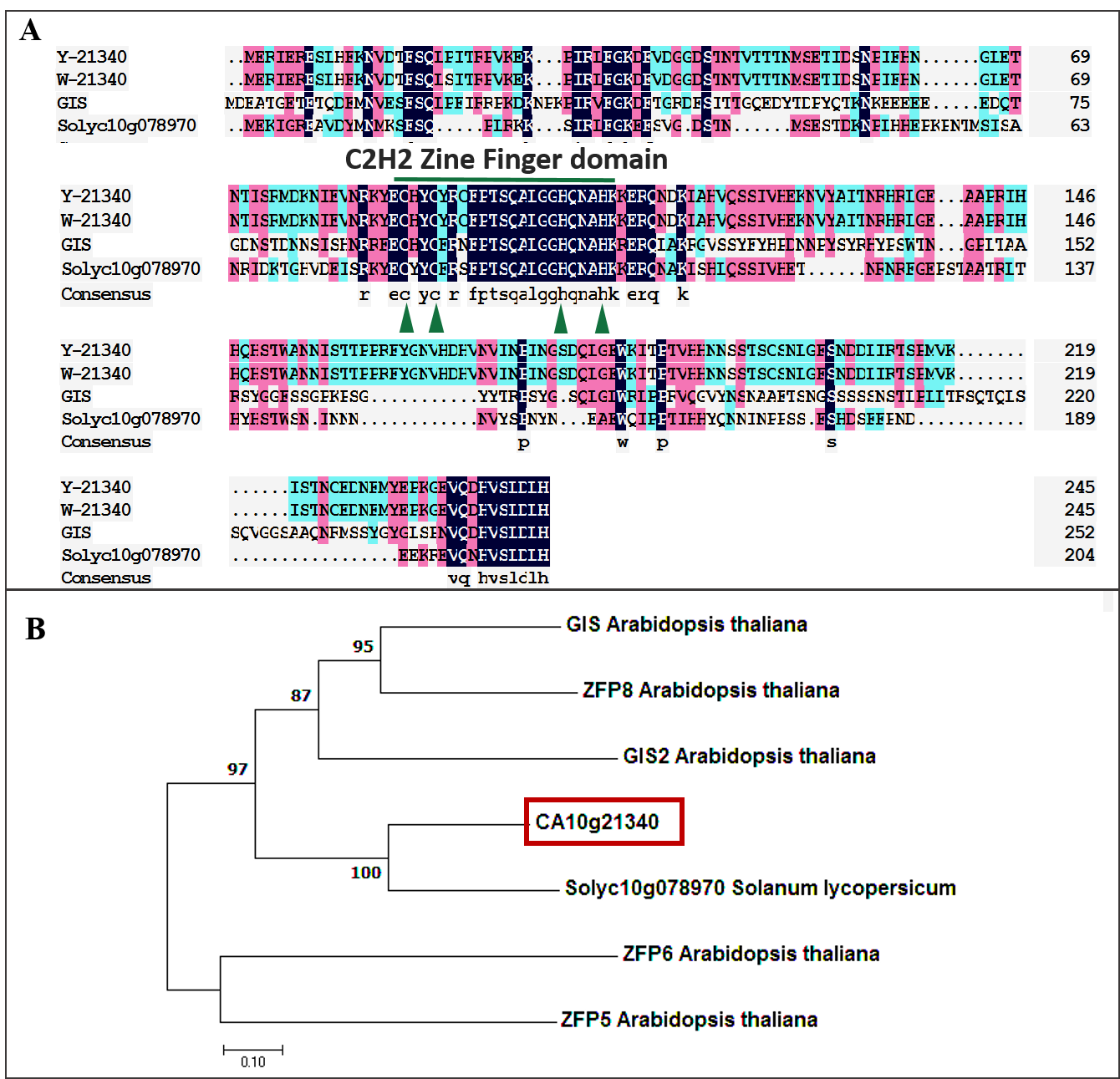
Supplementary Figure 2** Evolutionary analysis of annotation genes (**A)** Domain analysis of *CA10g21340*. (**B)** Phylogenetic tree analysis of C2H2 proteins

**
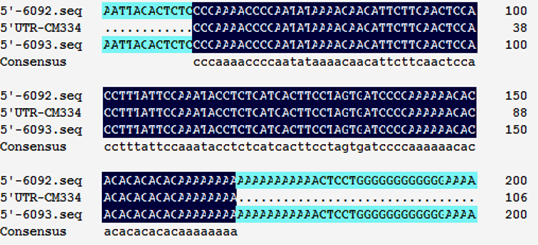
**

**Supplementary Figure 3** Sequence alignments of *CA10g21340* on 5’UTR region between hairiness line and hairless line

**
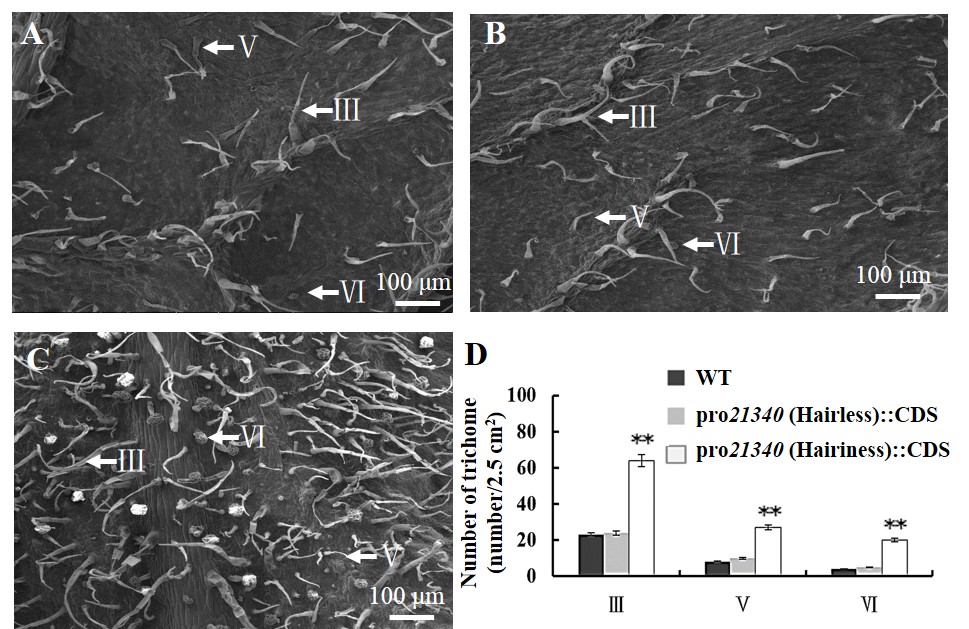
**

**Supplementary Figure 4 Trichome phenotypes and statistics of transgenic tomato. (A) Trichome phenotype of WT. (B) Trichome phenotype of *CA10g21340* gene driven by pro*21340* (Hairless) in Micro Tom. (C) Trichome phenotype of *CA10g21340* gene driven by pro*21340* (Hairiness) in Micro Tom. (D) Type and number of trichomes on leaves of WT and transgenic tomato**

**Supplementary Figures**

**Supplementary Table 1 Trichomes type and density of 280 pepper**

| No. | Species | Young leaves | Leaves | Young steam | Steam | Flower |
| --- | --- | --- | --- | --- | --- | --- |
| 17C603 | *C. annuum* | Ⅴ+Ⅵ+Ⅶ/2 | Ⅲ/2 | Ⅵ+Ⅶ/2 | - | Ⅷ+Ⅸ/2 |
| 17C604 | *C. annuum* | - | - | - | - | - |
| 17C605 | *C. annuum* | Ⅴ+Ⅵ+Ⅶ/1 | - | Ⅵ+Ⅶ/2 | - | Ⅷ+Ⅸ/1 |
| 17C607 | *C. annuum* | Ⅴ+Ⅵ+Ⅶ/2 | - | Ⅵ+Ⅶ/2 | - | Ⅷ+Ⅸ/2 |
| 17C609 | *C. annuum* | Ⅴ+Ⅵ+Ⅶ/2 | - | Ⅵ+Ⅶ/2 | - | Ⅷ+Ⅸ/2 |
| 17C611 | *C. annuum* | - | - | - | - | - |
| 17C613 | *C. annuum* | - | - | - | - | - |
| 17C614 | *C. annuum* | - | - | - | - | Ⅷ+Ⅸ/2 |
| 17C616 | *C. annuum* | - | - | Ⅱ+Ⅲ/2 | Ⅱ+Ⅲ/2 | Ⅷ+Ⅸ/1 |
| 17C627 | *C. annuum* | - | - | Ⅵ+Ⅶ/2 | - | - |
| 17C628 | *C. annuum* | Ⅴ+Ⅵ+Ⅶ/2 | - | Ⅵ+Ⅶ/2 | - | - |
| 17C629 | *C. annuum* | - | - | - | - | - |
| 17C630 | *C. annuum* | - | - | Ⅵ+Ⅶ/3 | - | Ⅷ+Ⅸ/2 |
| 17C632 | *C. annuum* | - | - | Ⅵ+Ⅶ/2 | - | Ⅷ+Ⅸ/2 |
| 17C634 | *C. annuum* | - | - | Ⅵ+Ⅶ/1 | - | Ⅷ+Ⅸ/1 |
| 17C638 | *C. annuum* | - | - | Ⅲ/2 | Ⅲ/1 | Ⅷ+Ⅸ/1 |
| 17C642 | *C. annuum* | - | - | Ⅵ+Ⅶ/2 | - | - |
| 17C648 | *C. annuum* | - | - | - | - | - |
| 17C650 | *C. annuum* | Ⅵ+Ⅶ/1 | - | Ⅵ+Ⅶ/2 | - | Ⅷ+Ⅸ/2 |
| 17C653 | *C. annuum* | Ⅴ+Ⅵ+Ⅶ/3 | - | Ⅵ+Ⅶ/1 | - | Ⅷ+Ⅸ/1 |
| 17C658 | *C. annuum* | Ⅴ+Ⅵ+Ⅶ/2 | - | Ⅵ+Ⅶ/3 | - | Ⅷ+Ⅸ/3 |
| 17C664 | *C. annuum* | Ⅴ+Ⅵ+Ⅶ/2 | Ⅴ/1 | Ⅱ+Ⅲ+Ⅵ+Ⅶ/3 | Ⅱ+Ⅲ/2 | Ⅷ+Ⅸ/2 |
| 17C668 | *C. annuum* | - | - | Ⅱ+Ⅲ/2 | Ⅱ+Ⅲ/2 | Ⅷ+Ⅸ/2 |
| 17C671 | *C. annuum* | - | - | - | - | - |
| 17C666 | *C. annuum* | Ⅵ+Ⅶ/2 | - | - | - | Ⅲ+Ⅷ+Ⅸ/2 |
| 17C667 | *C. annuum* | - | - | - | - | - |
| 17C675 | *C. annuum* | Ⅴ+Ⅵ+Ⅶ/1 | - | Ⅵ+Ⅶ/2 | - | Ⅷ+Ⅸ/2 |
| 17C678 | *C. annuum* | - | - | - | - | - |
| 17C682 | *C. annuum* | Ⅴ+Ⅵ+Ⅶ/2 | - | - | - | - |
| 17C689 | *C. annuum* | Ⅴ+Ⅵ+Ⅶ/2 | - | Ⅵ+Ⅶ/2 | - | Ⅷ+Ⅸ/2 |
| 17C690 | *C. annuum* | - | - | - | - | - |
| 17C691 | *C. annuum* | Ⅵ+Ⅶ/2 | - | Ⅵ+Ⅶ/2 | - | Ⅷ+Ⅸ/2 |
| 17C694 | *C. annuum* | Ⅵ+Ⅶ/2 | - | Ⅵ+Ⅶ/2 | - | Ⅷ+Ⅸ/2 |
| 17C695 | *C. annuum* | Ⅵ/1 | - | Ⅵ+Ⅶ/1 | - | Ⅷ+Ⅸ/1 |
| 17C697 | *C. annuum* | Ⅴ+Ⅵ+Ⅶ/2 | - | Ⅵ+Ⅶ/2 | - | Ⅷ+Ⅸ/2 |
| 17C698 | *C. annuum* | Ⅴ+Ⅵ+Ⅶ/2 | - | Ⅵ+Ⅶ/2 | - | Ⅷ+Ⅸ/2 |
| 17C702 | *C. annuum* | Ⅵ+Ⅶ/1 | - | Ⅵ+Ⅶ/2 | - | Ⅷ+Ⅸ/2 |
| 17C706 | *C. annuum* | - | - | Ⅵ+Ⅶ/1 | - | - |
| 17C710 | *C. annuum* | - | - | - | - | - |
| 17C711 | *C. annuum* | - | - | - | - | - |
| 17C713 | *C. annuum* | - | - | - | - | - |
| 17C714 | *C. annuum* | Ⅴ+Ⅵ+Ⅶ/2 | - | Ⅵ+Ⅶ/2 | - | Ⅷ+Ⅸ/2 |
| 17C715 | *C. annuum* | - | - | - | - | - |
| 17C718 | *C. annuum* | Ⅴ+Ⅵ+Ⅶ/2 | - | Ⅵ+Ⅶ/1 | - | Ⅷ+Ⅸ/2 |
| 17C720 | *C. annuum* | Ⅴ+Ⅵ+Ⅶ/2 | - | Ⅵ+Ⅶ/3 | - | Ⅷ+Ⅸ/3 |
| 17C723 | *C. annuum* | - | - | - | - | - |
| 17C726 | *C. annuum* | - | - | - | - | - |
| 17C734 | *C. annuum* | - | - | - | - | - |
| 17C733 | *C. annuum* | - | - | - | - | - |
| 17C737 | *C. annuum* | - | - | Ⅲ/2 | Ⅲ/1 | - |
| 17C739 | *C. annuum* | - | - | - | - | - |
| 17C740 | *C. annuum* | Ⅵ+Ⅶ/1 | - | Ⅵ+Ⅶ/1 | - | Ⅷ+Ⅸ/1 |
| 17C741 | *C. annuum* | - | - | Ⅵ+Ⅶ/2 | - | Ⅷ+Ⅸ/2 |
| 17C743 | *C. annuum* | Ⅴ/1 | - | Ⅵ+Ⅶ/2 | - | - |
| 17C742 | *C. annuum* | Ⅶ/2 | - | Ⅵ+Ⅶ/1 | - | Ⅷ+Ⅸ/2 |
| 17C751 | *C. annuum* | - | - | - | - | - |
| 17C744 | *C. annuum* | - | - | Ⅲ+Ⅵ+Ⅶ/2 | Ⅲ+Ⅵ+Ⅶ/2 | Ⅷ+Ⅸ/2 |
| 17C745 | *C. annuum* | Ⅴ+Ⅵ+Ⅶ/2 | - | Ⅵ+Ⅶ/2 | - | Ⅷ+Ⅸ/2 |
| 17C746 | *C. annuum* | - | - | - | - | - |
| 17C749 | *C. annuum* | - | - | - | - | - |
| 17C752 | *C. annuum* | - | - | - | - | - |
| 17C754 | *C. annuum* | - | - | - | - | - |
| 17C756 | *C. annuum* | - | - | Ⅱ+Ⅲ+Ⅵ+Ⅶ/2 | Ⅱ+Ⅲ+Ⅵ/2 | Ⅷ+Ⅸ/2 |
| 17C760 | *C. annuum* | Ⅴ+Ⅵ+Ⅶ/2 | - | Ⅵ+Ⅶ/2 | - | - |
| 17C761 | *C. annuum* | - | - | - | - | - |
| 17C762 | *C. annuum* | - | - | Ⅱ+Ⅲ+Ⅵ+Ⅶ/3 | Ⅱ+Ⅲ+Ⅵ/2 | Ⅷ+Ⅸ/2 |
| 17C763 | *C. annuum* | - | - | - | - | - |
| 17C764 | *C. annuum* | Ⅵ+Ⅶ/2 | - | Ⅵ+Ⅶ/2 | - | - |
| 17C773 | *C. annuum* | - | - | - | - | - |
| 17C774 | *C. annuum* | - | - | - | - | - |
| 17C776 | *C. annuum* | - | - | Ⅵ+Ⅶ/1 | - | - |
| 17C777 | *C. annuum* | - | - | Ⅱ+Ⅲ+Ⅵ+Ⅶ/2 | Ⅱ+Ⅲ+Ⅵ/2 | Ⅷ+Ⅸ/2 |
| 17C778 | *C. annuum* | Ⅴ+Ⅵ+Ⅶ/1 | - | Ⅵ+Ⅶ/2 | - | - |
| 17C779 | *C. annuum* | - | - | - | - | - |
| 17C780 | *C. annuum* | - | - | - | - | - |
| 17C781 | *C. annuum* | - | - | - | - | - |
| 17C122 | *C. annuum* | - | - | Ⅵ+Ⅶ/2 | - | Ⅷ+Ⅸ/2 |
| 17C782 | *C. annuum* | - | - | Ⅵ+Ⅶ/2 | - | - |
| 17C783 | *C. annuum* | Ⅴ+Ⅵ+Ⅶ/2 | - | Ⅵ+Ⅶ/2 | - | Ⅷ+Ⅸ/2 |
| 17C788 | *C. annuum* | Ⅴ+Ⅵ+Ⅶ/2 | - | Ⅵ+Ⅶ/2 | - | Ⅷ+Ⅸ/2 |
| 17C785 | *C. annuum* | - | - | Ⅵ+Ⅶ/2 | - | Ⅷ+Ⅸ/2 |
| 17C798 | *C. annuum* | Ⅴ+Ⅵ+Ⅶ/2 | - | Ⅵ+Ⅶ/2 | - | Ⅷ+Ⅸ/2 |
| 17C810 | *C. annuum* | - | - | Ⅵ+Ⅶ/2 | - | - |
| 17C812 | *C. annuum* | Ⅴ+Ⅵ/2 | - | Ⅵ+Ⅶ/2 | - | Ⅷ+Ⅸ/2 |
| 17C818 | *C. annuum* | - | - | - | - | - |
| 17C827 | *C. annuum* | Ⅴ+Ⅵ+Ⅶ/2 | - | Ⅵ+Ⅶ/2 | - | Ⅷ+Ⅸ/2 |
| 17C909 | *C. annuum* | Ⅴ+Ⅵ+Ⅶ/2 | - | Ⅵ+Ⅶ/2 | - | Ⅷ+Ⅸ/2 |
| 17C917 | *C. annuum* | - | - | - | - | - |
| 17C951 | *C. annuum* | - | - | - | - | Ⅷ+Ⅸ/1 |
| 17C958 | *C. annuum* | - | - | - | - | - |
| 17C962 | *C. annuum* | Ⅵ+Ⅶ/1 | - | Ⅵ+Ⅶ/1 | - | - |
| 17C965 | *C. annuum* | - | - | - | - | - |
| 17C967 | *C. annuum* | Ⅴ+Ⅵ+Ⅶ/2 | - | Ⅵ+Ⅶ/2 | - | Ⅷ+Ⅸ/2 |
| 17C969 | *C. annuum* | - | - | - | - | - |
| 17C970 | *C. annuum* | - | - | - | - | - |
| 17C977 | *C. annuum* | Ⅴ+Ⅵ/2 | - | Ⅵ+Ⅶ/2 | - | Ⅷ+Ⅸ/2 |
| 17C980 | *C. annuum* | Ⅴ+Ⅵ+Ⅶ/2 | - | Ⅵ+Ⅶ/2 | - | Ⅷ+Ⅸ/2 |
| 17C981 | *C. annuum* | Ⅴ+Ⅵ+Ⅶ/2 | - | Ⅵ+Ⅶ/2 | - | - |
| 17C983 | *C. annuum* | Ⅴ+Ⅵ+Ⅶ/2 | - | Ⅵ+Ⅶ/2 | - | - |
| 17C982 | *C. annuum* | Ⅴ+Ⅵ+Ⅶ/2 | - | Ⅵ+Ⅶ/2 | - | Ⅷ+Ⅸ/2 |
| 17C984 | *C. annuum* | Ⅴ+Ⅵ+Ⅶ/2 | - | Ⅵ+Ⅶ/2 | - | Ⅷ+Ⅸ/2 |
| 17C985 | *C. annuum* | - | - | - | - | - |
| 17C986 | *C. annuum* | - | - | - | - | - |
| 17C987 | *C. annuum* | - | - | - | - | - |
| 17C988 | *C. annuum* | - | - | - | - | - |
| 17C992 | *C. annuum* | - | - | - | - | - |
| 17C998 | *C. annuum* | Ⅴ+Ⅵ+Ⅶ/2 | - | Ⅵ+Ⅶ/2 | - | - |
| 17C1002 | *C. annuum* | Ⅴ/2 | Ⅴ/2 | Ⅵ+Ⅶ/2 | - | - |
| 17C1008 | *C. annuum* | Ⅴ+Ⅵ+Ⅶ/2 | - | Ⅵ+Ⅶ/2 | - | - |
| 17C1014 | *C. annuum* | Ⅴ+Ⅵ+Ⅶ/2 | - | Ⅵ+Ⅶ/2 | - | - |
| 17C1022 | *C. annuum* | Ⅵ+Ⅶ/2 | - | Ⅵ+Ⅶ/2 | - | - |
| 17C1028 | *C. annuum* | Ⅴ/2 | Ⅴ/2 | Ⅱ+Ⅲ+Ⅵ+Ⅶ/2 | Ⅱ+Ⅲ+Ⅵ+Ⅶ/2 | Ⅲ+Ⅴ+Ⅵ+Ⅸ/2 |
| 17C1032 | *C. annuum* | - | - | - | - | - |
| 17C1036 | *C. annuum* | - | - | - | - | - |
| 17C1037 | *C. annuum* | - | - | - | - | - |
| 17C1039 | *C. annuum* | Ⅴ+Ⅵ+Ⅶ/2 | - | Ⅱ+Ⅲ+Ⅵ+Ⅶ/2 | Ⅱ+Ⅲ+Ⅵ+Ⅶ/2 | Ⅷ+Ⅸ/2 |
| 17C1041 | *C. annuum* | - | - | - | - | - |
| 17C1061 | *C. annuum* | Ⅴ+Ⅵ+Ⅶ/2 | - | Ⅵ+Ⅶ/2 | - | Ⅷ+Ⅸ/2 |
| 17C1107 | *C. annuum* | Ⅴ+Ⅵ+Ⅶ/2 | - | Ⅱ+Ⅲ+Ⅵ+Ⅶ/3 | Ⅱ+Ⅲ+Ⅵ+Ⅶ/2 |  |
| 17C1109 | *C. annuum* | Ⅴ+Ⅵ+Ⅶ/2 | - | Ⅱ+Ⅲ+Ⅵ+Ⅶ/3 | Ⅱ+Ⅲ+Ⅵ+Ⅶ/2 | Ⅷ+Ⅸ/2 |
| 17C1110 | *C. annuum* | Ⅴ+Ⅵ+Ⅶ/2 | - | Ⅵ+Ⅶ/2 | - | - |
| 17C1111 | *C. annuum* | Ⅴ+Ⅵ+Ⅶ/2 | - | Ⅵ+Ⅶ/2 | - | Ⅷ+Ⅸ/2 |
| 17C1118 | *C. annuum* | - | - | - | - |  |
| 17C1113 | *C. annuum* | Ⅴ+Ⅵ+Ⅶ/2 | Ⅴ/1 | Ⅱ+Ⅲ+Ⅵ+Ⅶ/3 | Ⅱ+Ⅲ+Ⅵ+Ⅶ/2 | Ⅷ+Ⅸ/2 |
| 17C1114 | *C. annuum* | - | - | - | - | - |
| 17C1115 | *C. annuum* | - | - | - | - | Ⅷ+Ⅸ/2 |
| 17C1116 | *C. annuum* | Ⅴ+Ⅵ+Ⅶ/2 | Ⅴ/2 | Ⅱ+Ⅲ+Ⅵ+Ⅶ/3 | Ⅱ+Ⅲ+Ⅵ+Ⅶ/3 | Ⅲ+Ⅴ+Ⅵ+Ⅶ+Ⅷ+Ⅸ/2 |
| 17C1117 | *C. annuum* | Ⅴ+Ⅵ+Ⅶ/2 | - | Ⅵ+Ⅶ/2 |  | Ⅷ+Ⅸ/2 |
| 17C1119 | *C. annuum* | Ⅴ+Ⅶ/2 | Ⅴ/2 | Ⅱ+Ⅲ+Ⅵ+Ⅶ/2 | Ⅱ+Ⅲ+Ⅵ+Ⅶ/2 | Ⅴ+Ⅵ+Ⅶ+Ⅷ+Ⅸ/2 |
| 17C1120 | *C. annuum* | - | - | - | - | - |
| 17C1125 | *C. annuum* | Ⅵ+Ⅶ/2 | - | Ⅵ+Ⅶ/2 | - | - |
| 17C1127 | *C. annuum* | - | - | Ⅱ+Ⅲ+Ⅵ+Ⅶ/2 | Ⅱ+Ⅲ+Ⅵ+Ⅶ/2 | Ⅷ+Ⅸ/2 |
| 17C1133 | *C. annuum* | - | - | Ⅱ+Ⅲ+Ⅵ+Ⅶ/2 | Ⅱ+Ⅲ+Ⅵ+Ⅶ/2 | Ⅷ+Ⅸ/2 |
| 17C1140 | *C. annuum* | Ⅴ+Ⅵ+Ⅶ/2 | - | Ⅵ+Ⅶ/2 | - | - |
| 17C1144 | *C. annuum* | - | - | - | - | - |
| 17C1148 | *C. annuum* | - | - | - | - | - |
| 17C1151 | *C. annuum* | - | - | - | - | - |
| 17C1154 | *C. annuum* | - | - | - | - | - |
| 17C1155 | *C. annuum* | - | - | - | - | - |
| 17C1161 | *C. annuum* | - | - | - | - | - |
| 17C1166 | *C. annuum* | - | - | - | - | - |
| 17C1171 | *C. annuum* | - | - | - | - | - |
| 17C1175 | *C. annuum* | Ⅴ+Ⅵ/2 | - | - | - | - |
| 17C1176 | *C. annuum* | - | - | - | - | - |
| 17C1178 | *C. annuum* | Ⅴ+Ⅵ/2 | - | Ⅵ+Ⅶ/2 | - | Ⅷ+Ⅸ/2 |
| 17C1182 | *C. annuum* | - | - | - | - | - |
| 17C1183 | *C. annuum* | - | - | - | - | - |
| 17C1187 | *C. annuum* | Ⅴ+Ⅵ+Ⅶ/2 | Ⅴ/2 | Ⅱ+Ⅲ+Ⅵ+Ⅶ/3 | Ⅱ+Ⅲ+Ⅵ+Ⅶ/3 | Ⅲ+Ⅴ+Ⅷ+Ⅸ/2 |
| 17C1265 | *C. annuum* | - | - | - | - | - |
| 17C1275 | *C. annuum* | Ⅴ+Ⅵ+Ⅶ/2 | Ⅴ/2 | Ⅱ+Ⅲ+Ⅵ+Ⅶ/3 | Ⅱ+Ⅲ+Ⅵ+Ⅶ/3 | Ⅷ+Ⅸ/2 |
| 17C1212 | *C. annuum* | - | - | - | - | - |
| 17C1209 | *C. annuum* | Ⅴ+Ⅵ+Ⅶ/3 | Ⅴ/2 | Ⅱ+Ⅲ+Ⅵ+Ⅶ/3 | Ⅱ+Ⅲ+Ⅵ+Ⅶ/3 | Ⅲ+Ⅴ+Ⅷ+Ⅸ/2 |
| 17C1190 | *C. annuum* | Ⅴ+Ⅵ+Ⅶ/2 | - | Ⅵ+Ⅶ/2 | - | Ⅷ+Ⅸ/2 |
| 17C1192 | *C. annuum* | - | - | - | - | - |
| 17C1193 | *C. annuum* | - | - | - | - | - |
| 17C1194 | *C. annuum* | - | - | - | - | - |
| 17C1195 | *C. annuum* | Ⅲ+Ⅳ+Ⅴ+Ⅵ+Ⅶ/3 | Ⅲ+Ⅴ/2 | Ⅱ+Ⅲ+Ⅵ+Ⅶ/3 | Ⅱ+Ⅲ+Ⅵ+Ⅶ/3 | Ⅲ+Ⅴ+Ⅵ+Ⅶ+Ⅷ+Ⅸ/22 |
| 17C1196 | *C. annuum* | Ⅲ+Ⅴ+Ⅵ+Ⅶ/3 | Ⅲ+Ⅴ/2 | Ⅱ+Ⅲ+Ⅵ+Ⅶ/3 | Ⅱ+Ⅲ+Ⅵ+Ⅶ/3 | Ⅲ+Ⅴ+Ⅵ+Ⅶ+Ⅷ+Ⅸ/2 |
| 17C1197 | *C. chinensevs* | - | - | - | - | - |
| 17C1198 | *C. frutescens* | - | - | - | - | - |
| 17C1199 | *C. frutescens* | Ⅲ+Ⅳ+Ⅴ+Ⅵ+Ⅶ/2 | Ⅲ+Ⅴ+Ⅵ+Ⅶ/2 | Ⅱ+Ⅲ+Ⅵ+Ⅶ/2 | Ⅱ+Ⅲ+Ⅵ+Ⅶ/2 | Ⅲ+Ⅴ+Ⅵ+Ⅶ+Ⅷ+Ⅸ/2 |
| 17C1200 | *C. frutescens* | - | - | - | - | - |
| 17C1201 | *C. frutescens* | - | - | - | - | - |
| 17C1202 | *C. chinensevs* | - | - | - | - | - |
| 17C1203 | *C. chinensevs* | - | - | - | - | - |
| 17C1204 | *C. chinensevs* | - | - | - | - | - |
| 17C1205 | *C. baccatum* | Ⅲ+Ⅴ+Ⅵ+Ⅶ/3 | Ⅲ+Ⅴ+Ⅵ+Ⅶ/3 | Ⅱ+Ⅲ+Ⅵ+Ⅶ/3 | Ⅱ+Ⅲ+Ⅵ+Ⅶ/2 | Ⅲ+Ⅴ+Ⅵ+Ⅶ+Ⅷ+Ⅸ/2 |
| 17C1216 | *C. annuum* | Ⅴ+Ⅵ+Ⅶ/2 | - | Ⅵ+Ⅶ/2 | - | Ⅵ+Ⅶ+Ⅷ+Ⅸ/2 |
| 17C1217 | *C. annuum* | - | - | Ⅱ+Ⅲ+Ⅵ+Ⅶ/2 | Ⅱ+Ⅲ+Ⅵ+Ⅶ/2 | Ⅷ+Ⅸ/2 |
| 17C1220 | *C. annuum* | - | - | - | - | - |
| 17C1223 | *C. annuum* | Ⅴ+Ⅵ+Ⅶ/2 | - | Ⅱ+Ⅲ+Ⅵ+Ⅶ/3 | Ⅱ+Ⅲ+Ⅵ+Ⅶ/2 | Ⅷ+Ⅸ/2 |
| 17C1224 | *C. annuum* | Ⅴ/2 | Ⅴ/2 | Ⅱ+Ⅲ+Ⅵ+Ⅶ/3 | Ⅱ+Ⅲ+Ⅵ+Ⅶ/3 | Ⅶ+Ⅷ+Ⅸ/2 |
| 17C1225 | *C. annuum* | - | - | - | - | - |
| 17C1226 | *C. annuum* | - | - | Ⅱ+Ⅲ+Ⅵ+Ⅶ/2 | Ⅱ+Ⅲ+Ⅵ+Ⅶ/2 | Ⅷ+Ⅸ/2 |
| 17C1227 | *C. annuum* | - | - |  |  |  |
| 17C1228 | *C. annuum* | Ⅴ+Ⅶ/2 | - | Ⅱ+Ⅲ+Ⅵ+Ⅶ/2 | Ⅱ+Ⅲ+Ⅵ+Ⅶ/2 | Ⅷ+Ⅸ/2 |
| 17C1230 | *C. annuum* | Ⅴ+Ⅵ+Ⅶ/2 | Ⅴ/2 | Ⅱ+Ⅲ+Ⅵ+Ⅶ/2 | Ⅱ+Ⅲ+Ⅵ+Ⅶ/2 | Ⅴ+Ⅵ+Ⅶ/2 |
| 17C1231 | *C. annuum* | - | - | - | - | - |
| 17C1232 | *C. annuum* | - | - | Ⅵ+Ⅶ/2 | - | Ⅷ+Ⅸ/2 |
| 17C1792 | *C. annuum* | - | - | - | - | - |
| 17C1234 | *C. annuum* | - | - | - | - | - |
| 17C1235 | *C. annuum* | - | - | - | - | - |
| 17C1236 | *C. annuum* | - | - | - | - | - |
| 17C1237 | *C. annuum* | Ⅴ+Ⅵ+Ⅶ/2 | - | Ⅱ+Ⅲ+Ⅵ+Ⅶ/2 | Ⅱ+Ⅲ+Ⅵ+Ⅶ/2 | Ⅷ+Ⅸ/2 |
| 17C1238 | *C. annuum* | Ⅴ+Ⅵ+Ⅶ/2 | - | Ⅱ+Ⅲ+Ⅵ+Ⅶ/2 | Ⅱ+Ⅲ+Ⅵ+Ⅶ/2 | Ⅷ+Ⅸ/2 |
| 17C1239 | *C. annuum* | Ⅴ+Ⅵ+Ⅶ/3 | Ⅴ/2 | Ⅱ+Ⅲ+Ⅵ+Ⅶ/3 | Ⅱ+Ⅲ+Ⅵ+Ⅶ/3 | Ⅲ+Ⅴ+Ⅵ+Ⅶ+Ⅷ+Ⅸ/2 |
| 17C1240 | *C. annuum* | Ⅵ+Ⅶ/2 | - | Ⅱ+Ⅲ+Ⅵ+Ⅶ/2 | Ⅱ+Ⅲ+Ⅵ+Ⅶ/2 |  |
| 17C1241 | *C. annuum* | Ⅴ+Ⅵ+Ⅶ/2 | - | Ⅱ+Ⅲ+Ⅵ+Ⅶ/2 | Ⅱ+Ⅲ+Ⅵ+Ⅶ/2 | Ⅷ+Ⅸ/2 |
| 17C1242 | *C. annuum* | - | - | - | - | - |
| 17C1243 | *C. annuum* | Ⅴ+Ⅵ+Ⅶ/2 | - | Ⅱ+Ⅲ+Ⅵ+Ⅶ/2 | Ⅱ+Ⅲ+Ⅵ+Ⅶ/2 | Ⅷ+Ⅸ/2 |
| 17C1244 | *C. annuum* | Ⅴ+Ⅵ+Ⅶ/2 | - | Ⅵ+Ⅶ/2 | - | - |
| 17C1245 | *C. annuum* | Ⅴ/1 | - | Ⅵ+Ⅶ/1 | - | - |
| 17C1246 | *C. annuum* | - | - | - | - | - |
| 17C1247 | *C. annuum* | Ⅴ+Ⅵ+Ⅶ/2 | - | - | - | - |
| 17C1248 | *C. annuum* | Ⅲ+Ⅴ+Ⅵ+Ⅶ/3 | Ⅲ+Ⅴ/3 | Ⅱ+Ⅲ+Ⅵ+Ⅶ/3 | Ⅱ+Ⅲ+Ⅵ+Ⅶ/3 | Ⅲ+Ⅴ+Ⅵ+Ⅶ+Ⅷ+Ⅸ/2 |
| 17C1249 | *C. annuum* | Ⅲ+Ⅴ+Ⅵ+Ⅶ/3 | Ⅲ+Ⅴ/3 | Ⅱ+Ⅲ+Ⅵ+Ⅶ/3 | Ⅱ+Ⅲ+Ⅵ+Ⅶ/3 | Ⅲ+Ⅴ+Ⅵ+Ⅶ+Ⅷ+Ⅸ/2 |
| 17C1251 | *C. annuum* | Ⅲ+Ⅴ+Ⅵ+Ⅶ/3 | Ⅲ+Ⅴ/3 | Ⅱ+Ⅲ+Ⅵ+Ⅶ/3 | Ⅱ+Ⅲ+Ⅵ+Ⅶ/3 | Ⅲ+Ⅴ+Ⅵ+Ⅶ+Ⅷ+Ⅸ/2 |
| 17C1252 | *C. annuum* | - | - | Ⅱ+Ⅲ/2 | Ⅱ+Ⅲ/2 | - |
| 17C1254 | *C. annuum* | - | - | Ⅲ/2 | Ⅲ/2 | - |
| 17C1255 | *C. annuum* | - | - | Ⅱ+Ⅲ/2 | Ⅱ+Ⅲ/2 | - |
| 17C1256 | *C. annuum* | Ⅴ+Ⅵ+Ⅶ/2 | - | Ⅱ+Ⅲ+Ⅵ+Ⅶ/2 | Ⅱ+Ⅲ+Ⅵ+Ⅶ/2 | - |
| 17C1257 | *C. annuum* | Ⅲ+Ⅴ+Ⅵ+Ⅶ/3 | Ⅲ+Ⅴ/2 | Ⅱ+Ⅲ+Ⅵ+Ⅶ/3 | Ⅱ+Ⅲ+Ⅵ+Ⅶ/2 | Ⅲ+Ⅴ+Ⅵ+Ⅶ+Ⅷ+Ⅸ/2 |
| 17C1258 | *C. annuum* | - | - | - | - | - |
| 17C1259 | *C. annuum* | Ⅴ+Ⅵ+Ⅶ/2 | - | Ⅱ+Ⅲ+Ⅵ+Ⅶ/2 | Ⅱ+Ⅲ+Ⅵ+Ⅶ/2 | Ⅷ+Ⅸ/2 |
| 17C1260 | *C. annuum* | Ⅴ/2 | - | Ⅲ+Ⅴ/2 | Ⅲ+Ⅴ/2 | - |
| 17C1262 | *C. annuum* | - | - | - | - | - |
| 17C1263 | *C. annuum* | - | - | - | - | - |
| 17C1264 | *C. annuum* | - | - | - | - | - |
| 17C1266 | *C. annuum* | Ⅴ+Ⅵ+Ⅶ/2 | - | Ⅵ+Ⅶ/2 | - | - |
| 17C1267 | *C. annuum* | Ⅴ+Ⅵ+Ⅶ/2 | - | Ⅵ+Ⅶ/2 | - | - |
| 17C1268 | *C. annuum* | - | - | - | - | - |
| 17C1269 | *C. annuum* | Ⅴ+Ⅵ+Ⅶ/3 | Ⅲ+Ⅴ/3 | Ⅱ+Ⅲ+Ⅵ+Ⅶ/3 | Ⅱ+Ⅲ+Ⅵ+Ⅶ/3 | Ⅲ+Ⅴ+Ⅵ+Ⅶ+Ⅷ+Ⅸ/2 |
| 17C1270 | *C. annuum* | Ⅲ+Ⅴ/3 | Ⅲ+Ⅴ/3 | Ⅱ+Ⅲ+Ⅵ+Ⅶ/3 | Ⅱ+Ⅲ+Ⅵ+Ⅶ/3 | Ⅲ+Ⅴ+Ⅵ+Ⅶ+Ⅷ+Ⅸ/2 |
| 17C1271 | *C. annuum* | Ⅴ+Ⅵ+Ⅶ/2 | Ⅲ+Ⅴ/3 | Ⅱ+Ⅲ+Ⅵ+Ⅶ/3 | Ⅱ+Ⅲ+Ⅵ+Ⅶ/3 | Ⅲ+Ⅴ+Ⅵ+Ⅶ+Ⅷ+Ⅸ/2 |
| 17C1272 | *C. annuum* | Ⅴ+Ⅵ+Ⅶ/2 | - | Ⅱ+Ⅲ+Ⅵ+Ⅶ/3 | Ⅱ+Ⅲ+Ⅵ+Ⅶ/2 |  |
| 17C1273 | *C. annuum* | Ⅴ/2 | - | Ⅱ+Ⅲ+Ⅴ/2 | Ⅱ+Ⅲ+Ⅴ/2 | Ⅷ+Ⅸ/2 |
| 17C1274 | *C. annuum* | - | - | - | - | - |
| 17C1277 | *C. annuum* | Ⅵ+Ⅶ/2 | - | Ⅵ+Ⅶ/2 | - | - |
| 17C1278 | *C. annuum* | - | - | - | - | - |
| 17C1279 | *C. annuum* | - | - | - | - | - |
| 17C1280 | *C. annuum* | - | - | - | - | - |
| 17C1281 | *C. annuum* | Ⅴ+Ⅵ+Ⅶ/2 | - | Ⅵ+Ⅶ/2 | - | - |
| 17C1282 | *C. annuum* | - | - | - | - | - |
| 17C1283 | *C. annuum* | Ⅵ+Ⅶ/2 | - | Ⅵ+Ⅶ/2 | - | - |
| 17C1284 | *C. annuum* | - | - | - | - | - |
| 17C1288 | *C. annuum* | Ⅵ+Ⅶ/2 | - | Ⅵ+Ⅶ/2 | - | - |
| 17C1289 | *C. annuum* | Ⅴ+Ⅵ+Ⅶ/2 | - | Ⅱ+Ⅲ+Ⅴ/2 | Ⅱ+Ⅲ+Ⅴ/2 | Ⅷ+Ⅸ/2 |
| 17C1290 | *C. annuum* | Ⅴ/2 | - | Ⅲ+Ⅴ/2 | Ⅲ+Ⅴ/2 | Ⅷ+Ⅸ/2 |
| 17C1291 | *C. annuum* | Ⅴ+Ⅵ+Ⅶ/2 | - | Ⅲ+Ⅴ/2 | Ⅲ+Ⅴ/2 | Ⅷ+Ⅸ/2 |
| 17C1292 | *C. annuum* | - | - | - | - | - |
| 17C1293 | *C. annuum* | - | - | - | - | - |
| 17C1294 | *C. annuum* | - | - | - | - | - |
| 17C1295 | *C. annuum* | - | - | - | - | - |
| 17C1296 | *C. annuum* | Ⅴ+Ⅵ+Ⅶ/2 | - | Ⅵ+Ⅶ/2 | - | - |
| 17C1297 | *C. annuum* | Ⅵ+Ⅶ/2 | - | Ⅵ+Ⅶ/2 | - | - |
| 17C1298 | *C. annuum* | Ⅵ+Ⅶ/2 | - | Ⅵ+Ⅶ/2 | - | - |
| 17C1299 | *C. annuum* | Ⅴ+Ⅵ+Ⅶ/2 | - | Ⅵ+Ⅶ/2 | - | - |
| 17C1300 | *C. annuum* | Ⅲ+Ⅴ+Ⅵ+Ⅶ/2 | - | Ⅱ+Ⅲ+Ⅵ+Ⅶ/3 | Ⅱ+Ⅲ+Ⅵ+Ⅶ/3 | Ⅷ+Ⅸ/2 |
| 17C1301 | *C. annuum* | Ⅲ+Ⅴ+Ⅵ+Ⅶ/2 | - | Ⅱ+Ⅲ+Ⅵ+Ⅶ/3 | Ⅱ+Ⅲ+Ⅵ+Ⅶ/3 | Ⅷ+Ⅸ/2 |
| 17C1302 | *C. annuum* | Ⅲ+Ⅴ+Ⅵ+Ⅶ/2 | - | Ⅱ+Ⅲ+Ⅵ+Ⅶ/2 | Ⅱ+Ⅲ+Ⅵ+Ⅶ/1 | Ⅷ+Ⅸ/2 |
| 17C1303 | *C. annuum* | Ⅴ/2 | - | Ⅵ+Ⅶ/2 | - | - |
| 17C1304 | *C. annuum* | - | - | - | - | - |
| 17C1305 | *C. annuum* | Ⅵ+Ⅶ/2 | - | Ⅵ+Ⅶ/2 | - | - |
| 17C1306 | *C. annuum* | - | - | - | - | - |
| 17C1307 | *C. annuum* | - | - | - | - | - |
| 17C1308 | *C. annuum* | - | - | - | - | - |
| 17C1309 | *C. annuum* | Ⅴ+Ⅵ+Ⅶ/2 | - | Ⅵ+Ⅶ/2 | - | - |
| 17C1310 | *C. annuum* | - | - | - | - | - |
| 17C1311 | *C. annuum* | Ⅴ+Ⅵ+Ⅶ/2 | - | Ⅵ+Ⅶ/2 | - | - |
| 17C1312 | *C. annuum* | Ⅴ+Ⅵ+Ⅶ/2 | - | Ⅵ+Ⅶ/2 | - | - |
| 17C1313 | *C. annuum* | Ⅴ+Ⅵ+Ⅶ/2 | Ⅴ/2 | Ⅱ+Ⅲ+Ⅵ+Ⅶ/3 | Ⅱ+Ⅲ/3 | Ⅲ+Ⅴ+Ⅷ+Ⅸ/2 |
| 17C1314 | *C. annuum* | - | - |  | - | - |
| 17C1315 | *C. annuum* | Ⅴ+Ⅵ+Ⅶ/2 | - | Ⅵ+Ⅶ/2 | - | - |
| 17C1316 | *C. annuum* | - | - |  | - | - |
| 17C1317 | *C. annuum* | Ⅵ+Ⅶ/2 | - | Ⅵ+Ⅶ/2 | - | - |
| 17C1318 | *C. annuum* | Ⅵ+Ⅶ/2 | - | Ⅵ+Ⅶ/2 | - | - |
| 17C1319 | *C. annuum* | Ⅴ+Ⅵ+Ⅶ/2 | - | Ⅵ+Ⅶ/2 | - | - |
| 17C1320 | *C. annuum* | Ⅲ+Ⅴ+Ⅵ+Ⅶ/2 | - | Ⅲ+Ⅴ+Ⅵ+Ⅶ/3 | Ⅲ+Ⅴ+Ⅶ/3 | Ⅴ+Ⅵ+Ⅶ/2 |
| 17C1321 | *C. annuum* | Ⅵ+Ⅶ/2 | - | Ⅵ+Ⅶ/2 | - | - |
| 17C1322 | *C. annuum* | - | - | - | - | - |
| 17C1323 | *C. annuum* | - | - | - | - | - |
| 17C1324 | *C. annuum* | Ⅴ/2 | - | Ⅵ+Ⅶ/2 | - | - |
| 17C1325 | *C. annuum* | Ⅵ+Ⅶ/2 | - | Ⅵ+Ⅶ/2 | - | - |
| 17C1326 | *C. annuum* | Ⅴ+Ⅵ+Ⅶ/2 | - | Ⅵ+Ⅶ/2 | - | - |
| 17C1327 | *C. annuum* | - | - |  | - | - |
| 17C1328 | *C. annuum* | Ⅵ+Ⅶ/2 | - | Ⅵ+Ⅶ/2 | - | - |
| 17C1329 | *C. annuum* | - | - |  | - | - |
| 17C1330 | *C. annuum* | Ⅴ+Ⅵ+Ⅶ/2 | - | Ⅵ+Ⅶ/2 | - | - |
| 17C1331 | *C. annuum* | - | - | - | - | - |
| 17C1332 | *C. annuum* | - | - | - | - | - |
| 17C1730 | *C. annuum* | - | - | - | - | - |
| 17C1826 | *C. annuum* | Ⅴ+Ⅵ+Ⅶ/3 | Ⅴ/3 | Ⅲ+Ⅴ+Ⅵ+Ⅶ/3 | Ⅲ+Ⅴ/3 | Ⅲ+Ⅴ+Ⅷ+Ⅸ/2 |
| 17C1830 | *C. annuum* | - | - | - | - | - |
| 17C1996 | *C. annuum* | - | - | - | - | - |
| 17C2059 | *C. annuum* | Ⅴ+Ⅵ+Ⅶ/2 | - | Ⅵ+Ⅶ/2 | - | - |
| 17C466 | *C. annuum* | - | - | - | - | - |
| 17C469 | *C. annuum* | - | - | - | - | - |
| 17C470 | *C. annuum* | Ⅵ+Ⅶ/2 | - | Ⅵ+Ⅶ/2 | - | - |
| 17C471 | *C. annuum* | - | - | - | - | - |
| 17C472 | *C. annuum* | - | - | - | - | - |

**Note:** Ⅱ-Ⅸ showed types of pepper trichomes. 4 showed abundant (800-1500 gen/cm^2^), 3 showed medium (200-800 gen/cm^2^), 2 showed sparse (1-200 gen/cm^2^) and “-” showed no trichomes

**Supplementary Table 2 Genetic analysis of *Hairiness* gene**

| Population | Hairiness individuals | Hairless individuals | Segregation ration | χ² value |
| --- | --- | --- | --- | --- |
| F_1_（2018） | 72 | 0 | - | - |
| F_2_（18C2480） | 510 | 165 | 3:1 | 0.862 |
| F_2_（19Q6090） | 649 | 220 | 3:1 | 0.923 |

**Supplementary Table 3 Genotypes and phenotypes of F_3_ exchange individuals**

| F_3_ individuals | Number  of plants | Phenotype | Markers | | | | | | |
| --- | --- | --- | --- | --- | --- | --- | --- | --- | --- |
|  |  |  | In2323 | HpmE031 | Ind21340-2 | In21380 | Ind21430 | In21440-1 | Ind440 |
| 766 | 6 | c | b | b | b | a | a | a | a |
| 330 | 5 | a | b | b | b | a | a | a | a |
| 663 | 5 | c | a | a | a | b | b | b | b |
| 587 | 4 | a | a | a | a | b | b | b | b |
| 586 | 5 | c | a | a | a | b | b | b | b |

**Supplementary Table 4 Annotated genes in the mapping region**

| Code | Gene | Position | Annotation |
| --- | --- | --- | --- |
| ORF1 | *CA10g21340* | 231754268..231755974 | Zinc Finger Protein |
| ORF2 | *CA10g21350* | 231790909..231791394 | Unknown protein |
| ORF3 | *CA10g21360* | 231793990..231794589 | Unknown protein |
| ORF4 | *CA10g21370* | 231813482..231815719 | Unknown protein |

**Supplementary Table 5 Cis-acting elements in the *CA10g21340* promoter regions**

| Hairiness plant | Cis-acting element | Hairless plant | Site | Sequences |
| --- | --- | --- | --- | --- |
| 1 | ABRE: abscisic acid | 1 |  |  |
| 3 | ERE: ethylene-responsive | 4 | 99 | ATTTTAAA |
| 1 | TATC-box: gibberellin-responsiveness | 1 |  |  |
| 1 | TCA-element: salicylic acid -responsiveness | 1 |  |  |
| 1 | 3-AF1 binding site | 1 |  |  |
| 6 | Box 4 | 5 | 402 | ATTAAT |
| 3 | GATA-motif | 3 |  |  |
| 1 | G-Box | 1 |  |  |
| 1 | W box | 1 |  |  |
| 1 | ARE | 1 |  |  |
| 2 | MBS | 2 |  |  |
| 3 | MYB | 2 | 1055 | CAACAG |
| 3 | MYC | 4 | 1854 | CATTTG |
| 2 | Myb | 2 |  |  |
| 1 | WRE3 | 1 |  |  |
| 1 | STRE | 1 |  |  |
| 2 | AAGAA-motif | 1 | 1097 | GAAAGAA |
| 1 | AC-II (adenylyl cyclase ) | 1 |  |  |
| 1 | AT-rich element | 1 |  |  |
| - | AT-rich sequence | 1 | 2060 | ATAGAAATCAA |
| 16 | AT~TATA-box | 21 |  |  |
| 47 | CAAT-box | 46 |  |  |
| 1 | O_2_-site | 1 |  |  |
| 77 | TATA-box | 100 |  |  |
| 1 | circadian | 1 |  |  |

**Supplementary Table 6 Information of InDel markers in fine mapping of *Hairiness* gene**

| Marker | Chromosome | Forward | Reverse |
| --- | --- | --- | --- |
| SR096 | 227990526 | TTACATAGAAGGCACCAGGC | GGTGGGTGCGGAATTCTT |
| SR214 | 228003591 | ACCTTGAACCGGGGGTCT | GCCGAAACTGTTGGACTCC |
| SR324 | 228352733 | TCCCCGCAACTTAGAATAGCA | ACGAGACACAACAGTCAACG |
| SR470 | 228723210 | TTGAGGGACCGGTTTCCT | TTGAGGGACCGGTTTCCT |
| SR519 | 230188529 | TGGACTGGTTGACCCAAACA | AGACGTTGAACCTCCGTTGA |
| SR660 | 231398729 | GAATCCTACATGGTGCAAGCT | CCACATGAACCCAACGAGC |
| SR711 | 231992485 | TCAGAGCATTCAACTTGCACT | ACACCTCACATTTTGTCGGTG |
| SR724 | 232402623 | ACCATCAACCTTCGTGCTCT | TGCATGGTGAACCCTTTTGA |
| SR890 | 232132324 | CTCACACCAGCACAGCCA | CACCGAATGGGGCCCTAC |
| SRlj60 | 231462904 | TTTGGCAACTCAGTCTCGTG | TGTTTCGGTTAGACCCTTGG |
| SRlj1012 | 232373643 | TCTTCAGAGCTTGCTACATAC | GGGACCCACTAAGGATAAA |
| HpmE031 | 231723402 | CCCTAAATCAACCCCAAATT | CCCCCATTACCTGACTGCAA |
| In10-6 | 230844000 | AATCATAATCAAACAGCCTAACG | GATAATATCACTATCTCGGGGAG |
| In10-7 | 230847257 | TAACTTGTAAGATTTCGCTGGAT | GCCATAACTTTTGACTCGTTGAT |
| In10-8 | 231096036 | TATCCGAGCGAAGGAGGGCGTAG | TTCTTGTCAAACTCAAGCCATCA |
| In10-9 | 231753172 | CTGCAGTTTGATGCATTAAAGCT | TATTTGGGCCATTATTTGAGACA |
| In2423 | 231647710 | AAGTTTTCAATATGACAATACTC | AGAAATAGACTCTTTTAAGTTCA |
| Ind440 | 231900189 | TTAGCTTGGTTGCTTTAATTCTG | ACCCCACTACAAAGGTGTATCTT |
| Ind450 | 231931515 | TTCCTTCTATTTCAATTAAAGTGG | ACTTGATTTCTTAGATGATGCCA |
| InP2434 | 231941966 | CCCTATTTTGTGTCACGCATTAT | CAGACGTAATCCTACACACTAAC |
| In21340-2 | 231753172 | CTTATTACCGACAGTTGTTCAGC | CTCTTATTCGTTCGGCCTACCAT |
| In21380 | 231822140 | TTTCATTGTATTACTAGGGAGTT | AAAGGGAAGCTGATACATTAAAG |
| In21430 | 231876286 | ATTTCATTGACAAGAACGTTTTATG | ACTTTCGGACGCACTTGATTCTA |
| In21440 | 231900189 | TTAGCTTGGTTGCTTTAATTCTG | ACCCCACTACAAAGGTGTATCTT |

**Supplementary Table 7 Information of primers used to develop constructs and qPCR of *Hairiness* gene**

| Primer | Sequence（5'-3'） |
| --- | --- |
| pTRV2-21340 | CGCGGATCCAGTGCATGAGAAGAACGTTTACGC  CGGGGTACCCATGATCTTGAACTTCTCCTTTTGG |
| pTOPO-TA-21340 Hairiness/Hairless | ATGGAAAGGATTGAAAGAGAATCTC  TTACAAGTGCAAATCTAAACTCACATG |
| pTOPO-TA-Pro21340 Hairiness/Hairless | ACATTACTGAGCTAGGCGTCTGATA  AGAGGTATTTGGAATAAAGGTGGAG |
| pTOPO-TA-5’UTR Hairiness/Hairless | TAGCTCCAATACAGGTATCGAACATCA  GAAGGTATCCACATTCTTGAAATGAAG |
| p1305-Pro2288 | F: GAGCTCGGTACCCGGGGATCCACATTACTGAGCTAGGCGTCTGATA  R: GAAATTTACCCTCAGATCTACCATGGAGAGGTATTTGGAATAAAGGT |
| p1305-Pro1690 | F: GAGCTCGGTACCCGGGGATCCTACCCTCAGATCTACCATGGGTAATT  R：Consistent with p1305-Pro2288 |
| p1305-Pro1108 | F: GAGCTCGGTACCCGGGGATCCTGTACGTATATGTGCAGAGTGGTTT  R：Consistent with p1305-Pro2288 |
| p1305-Pro582 | F: GAGCTCGGTACCCGGGATCCTAGCTAAATATGAAGATTTGGCTAT  R：Consistent with p1305-Pro2288 |
| p1305-Pro324 | F: GAGCTCGGTACCCGGGGATCCCTGGGTATGAAAAAATCCTTGATAT  R：Consistent with p1305-Pro2288 |
| p1305-Pro240 | F: GAGCTCGGTACCCGGGGATCCCAATACAGGTATCGAACATCAATGG  R：Consistent with p1305-Pro2288 |
| p1305-Pro1608 to 1120 | F:GAGCTCGGTACCCGGGGATCCGATTGCAGTTGTTAATCATTTAAGG  R: GAAATTTACCCTCAGATCTACCATGGCATCAAAGAATTCTTTTAGTA |
| p1305-Pro1043 to 628 | F: GAGCTCGGTACCCGGGGATCCAAAACTGTGAGCTACAACAACTTTG  R:GAAATTTACCCTCAGATCTACCATGGATGTTTACAACGATAGAAACG |
| p1305-Pro650 to 349 | F: GAGCTCGGTACCCGGGGATCCCACGTTTCTATCGTTGTAAACATCC  R: GAAATTTACCCTCAGATCTACCATGGGACCTTTGATTCCGATCAAGA |
| p1305-Pro582 to -218 | F: GAAATTTACCCTCAGATCTACCATGGGTAATTGGTCTTTTTTTGGGG  R: GAGCTCGGTACCCGGGGATCCAGAGGTATTTGGAATAAAGGTGG |
| p1305-Pro2288(Hairiness/Hairless)-21340(Hairiness) | F:GAAATTTACCCTCAGATCTACCATGGTTACAAGTGCAAATCTAAACT  R:CTAGAAATTTACCCTCAGATCTTTACAAGTGCAAATCTAAACTCAC |
| q*CA10g21340* | F: TACGAACACTGTGACCACCA  R: TGAGAAGTTGGGAAGCACCTA |
| *Actin*  (*Solyc11g005330*) | F: GCAGGTATCCACGAGACT  R: CCACCACTGAGCACAATG |
| *UIB-3*  (*Capana06g002873*) | F: TGTCCATCTGCTCTCTGTTG  R: CACCCCAAGCACAATAAGAC |
